# Effects of lifestyle physical activity and sedentary behaviour interventions on disease activity and patient- and clinician- important health outcomes in rheumatoid arthritis: a systematic review with meta-analysis

**DOI:** 10.1186/s41927-023-00352-9

**Published:** 2023-09-06

**Authors:** Sophia M. Brady, Jet J. C. S. Veldhuijzen van Zanten, Petros C. Dinas, Tom E. Nightingale, George S. Metsios, Saleh M. A. Elmsmari, Joan L. Duda, George D. Kitas, Sally A. M. Fenton

**Affiliations:** 1 School of Sport, Exercise and Rehabilitation Sciences, University of Birmingham, Birmingham, B15 2TT UK; 2 Rheumatology Department, Dudley Group NHS Foundation Trust, Dudley, UK; 3 Medical Research Council- Versus Arthritis Centre for Musculoskeletal Ageing, University of Birmingham, Birmingham, UK; 4 FAME Laboratory, Department of Physical Education and Sport Science, University of Thessaly, Thessaly, Greece; 5 Centre for Trauma Science Research, University of Birmingham, Birmingham, UK; 6 Department of Nutrition and Dietetics, School of Physical Education, Sport Science and Dietetics, University of Thessaly, Thessaly, Greece; 7 Institute of Mental Health, University of Birmingham, Birmingham, UK

**Keywords:** Rheumatoid arthritis, Systematic review, Physical activity, Sedentary behaviour, Intervention, Lifestyle physical activity, Health, Disease activity

## Abstract

**Background:**

Lifestyle physical activity (PA) is defined as any type of PA undertaken as part of daily life. It can include engagement in activities of daily living (i.e., household chores, gardening, walking to work), incidental PA, walking and/or reducing sedentary or sitting behaviours (SB). Regular PA is recommended for people with Rheumatoid Arthritis (RA) to reduce disease activity and systemic inflammation, as well as to improve patient- and clinician-important health outcomes. However, there is no summarised evidence of the effectiveness of interventions specifically targeting lifestyle PA and SB in this population. The aims of this systematic review with meta-analysis were to evaluate interventions targeting lifestyle PA and/or SB on 1) disease activity; 2) PA, SB and 3) patient- and clinician-important outcomes in people with RA.

**Methods:**

Eight databases [Medline, Cochrane Library CENTRAL, Web of Science, PsychINFO, Cumulative Index to Nursing & Allied Health Literature, Scopus, Excerpta Medica database and Physiotherapy Evidence Database] were searched from inception-August 2022. Inclusion criteria required interventions to target lifestyle PA and/or SB, conducted in adults with RA, assessing patient- and/or clinician-important outcomes.

**Results:**

Of 880 relevant articles, 16 interventions met the inclusion criteria. Meta-analyses showed statistically significant effects of interventions on disease activity (standardised mean difference = -0.12 (95% confidence interval = -0.23 to -0.01, I^2^ = 6%, z = 2.19, *p* = .03), moderate-to-vigorous PA, light/leisure PA, steps, functional ability, and fatigue. Whereas, no intervention effects were visualised for total PA, pain, anxiety or quality of life.

**Conclusions:**

Lifestyle PA interventions led to increased PA, reductions in SB and improvements in disease activity and other patient- and/or clinician-important health outcomes in people with RA. Future interventions should be less heterogenous in content, structure, focus and outcome measures used to aid understanding of the most effective intervention components for improving health. More SB interventions are needed to determine their effectiveness at producing clinical benefits.

**Supplementary Information:**

The online version contains supplementary material available at 10.1186/s41927-023-00352-9.

## Key points

• Lifestyle physical activity and sedentary behaviour interventions are effective at improving disease activity, increasing physical activity and improving some patient- and clinician- important outcomes, in people with Rheumatoid Arthritis.

• Few interventions conducted follow-up assessments of outcomes, making it difficult to determine if changes in response to interventions were sustained beyond the intervention itself.

• The lack of beneficial effect of interventions on some patient- and/or clinician-important outcomes may be due to heterogeneity in intervention length, content and method of outcome assessment. This highlights the need for a consensus on optimal measurement methods and reporting for health outcomes, for interventions effects to be reliably and accurately evaluated.

• There is a need for more consistency in lifestyle physical activity and sedentary behaviour interventions, to determine what intervention components are most effective at improving health in people with Rheumatoid Arthritis.

**Supplementary Information:**

The online version contains supplementary material available at 10.1186/s41927-023-00352-9.

## Background

Rheumatoid Arthritis (RA) is a chronic inflammatory autoimmune condition, characterised by high levels of pain and fatigue [1, 2]. Consequently, people with RA frequently report low levels of physical activity (PA), with a significant proportion of daily life engaged in sedentary behaviours (SB) [3–5]. PA is defined as any bodily movement produced by skeletal muscles that leads to an energy expenditure beyond the resting rate, and SB is defined as any waking activity expending energy ≤ 1.5 metabolic equivalents (METs) whilst in a sitting/reclining/lying posture [6]. In people with RA, participating in PA has shown reductions in disease activity and markers of systemic inflammation, and improvements in functional ability, pain, fatigue, depression and anxiety [7–11]. Therefore, regular PA, as well as self-management, is recommended as a non-pharmacological approach in RA [12]. Furthermore, recent evidence has shown that high levels of SB are independently related to increased disease activity, reduced functional ability and pain in people with RA [13–15]. Together, the independent health benefits of PA and SB emphasise the need for behavioural interventions to encourage PA and/or reduce SB in people with RA.

Previously, the most commonplace non-pharmacological interventions in RA involved structured, supervised, and purposeful exercise, targeting moderate-to-vigorous PA (MVPA) (i.e., behaviour ≥ 3 METs) [7, 16]. Despite the well-known benefits of MVPA, exercise training can be difficult for people with RA, especially in those with high disease activity [13] who experience additional barriers to being active [17]. In addition, many studies misreport information about the “dose” of exercise (i.e., frequency, intensity, time and type of exercise, and training principles), limiting the clarity, accuracy and reproducibility of results [18]. Consequently, alternative therapeutic approaches and interventions that focus on increasing overall PA, through incorporating more PA into an individual’s daily lifestyle, are increasingly advocated [19]. This approach of increasing “lifestyle PA”, may be perceived as more feasible, achievable, and sustainable for people with RA [20].

Although there is no formal definition for lifestyle PA, it comprises increasing any type of PA as part of day-to-day life. This can include increasing engagement in activities of daily living (e.g., gardening, housework, walking to work), increasing incidental PA (i.e., PA built up in small amounts over the day), as well as increasing engagement in activities such as walking. Reducing SB is also an avenue to increasing lifestyle PA, as sitting less will assist in increasing an individual’s total daily PA, irrespective of intensity [21]. In healthy individuals and amongst those living with other musculoskeletal conditions, emerging evidence has suggested that engagement in lifestyle PA is a clinically meaningful and cost-effective strategy to increase PA and improve health outcomes, with good compliance and high acceptability [21–25].

There is little summarised and synthesised evidence regarding the effectiveness of lifestyle PA and SB interventions in people with RA, particularly related to improving core patient- and clinician-important outcomes (i.e., outlined by Outcome Measures in Rheumatoid Arthritis Clinical Trials, OMERACT), and particularly disease activity. Disease activity is associated with disease progression, severity, hospitalisation and comorbidities in RA [7, 26]. There is substantial evidence that exercise interventions can reduce disease activity [27]. However, to our knowledge, no systematic review has assessed the effectiveness of lifestyle PA and SB interventions at improving disease activity in the RA population. To understand the value of lifestyle interventions to promote PA or reduce SB for improving health outcomes in RA, it is important to examine and appraise the current evidence. The aim of this systematic review with meta-analysis was to evaluate the effectiveness of lifestyle PA and SB (both individually and collectively) interventions on disease activity, PA and SB engagement, and other core OMERACT patient- and clinician-important outcomes in people with RA [28, 29].

## Methods

### Registration

This systematic review was registered in the International Prospective Register of Systematic Review database (PROSPERO, CRD42020149345).

### Electronic data sources and literature searches

Following the Preferred Reporting Items for Systematic Reviews and Meta-Analyses (PRISMA) guidelines [30] and the Cochrane Handbook [31], a literature search strategy was designed, through consultations with research librarians and members of the research team (GM, SF and JVvZ). The PICO method was used to assist search strategy creation (Supplementary Table 1), and the search strategy was adapted for each database.

Eight databases [Medline, Cochrane Library CENTRAL, Web of Science, PsychINFO, Cumulative Index to Nursing & Allied Health Literature (CINAHL), Scopus, Excerpta Medica database (EMBASE) and Physiotherapy Evidence Database (PEDro)] were searched from inception to August 2022 to identify relevant publications. The search algorithms used in each database can be found in Supplementary Table 2.

### Study selection and inclusion criteria

Two review team members reviewed and selected the eligible publications to be included in the systematic review, independently (SB and SE) for both title and abstract and full text screening. A third review member acted as a referee (JVvZ) to resolve any conflict between the investigators who performed the selection process. Where title and abstract did not provide sufficient information regarding the intervention, full texts were examined. Reference lists of included articles were manually examined to supplement searches and identify further relevant studies.

In order to be considered for inclusion, studies needed to: 1) be conducted in adults (≥ 18 years) with self- or physician-diagnosis of RA; 2) include an intervention of any length which is directly or indirectly targeting lifestyle PA and/or SB; 3) include assessments of core patient- (i.e., functional ability, pain, fatigue, depression, anxiety, vitality, quality of life) and/or clinician- (i.e., disease activity, functional ability) important outcomes, as defined by OMERACT [32–34]; and 4) include an outcome measure quantifying lifestyle PA and/or SB, such as pedometer-assessed daily steps, self-reported total daily PA, or accelerometer-assessed MVPA. Publications were also required to be in English, with no restrictions on length of follow-up or geographic location. Randomised controlled trials (RCTs), quasi-randomised and single-arm trials were included in this review. Studies involving participants with various diagnoses of arthritis, whereby the results of RA participants could not be distinguished from other cohorts (e.g., osteoarthritis), were excluded. Multi-component interventions (i.e., which focused on other behaviours alongside PA, such as diet), were included if they; 1) included a component focused on lifestyle PA and/or SB, and 2) measured PA and/or SB as an outcome. This will provide novel insight regarding the relative success of interventions primarily focused on increasing PA/reducing SB vs. to multi-component interventions in which promoting PA/reducing SB is not the only aim. Protocols, review articles, poster presentations and conference proceedings were also excluded.

The primary outcome in this review was disease activity, as it is a OMERACT patient- and clinician-important outcome, a key clinical target for treatment and management of RA, and a predictor of health, disease severity and hospitalisation [7, 26, 35]. Secondary outcomes consisted of lifestyle PA and SB (including, total PA, steps, MVPA, and leisure/light intensity PA and sedentary time) and other core patient and/or clinician important outcomes relevant to RA (pain, functional ability, fatigue, anxiety, depression and quality of life) [28, 29].

### Data extraction and risk of bias assessment

Data were extracted from all included studies, by two independent review team members (SB and SE). Details of each study were collected and characterised by author, date of publication, sample size, participant characteristics (i.e., age, gender, disease duration, and disease activity), intervention characteristics (i.e., description of intervention, assessment timepoints and intervention length), methods of outcome assessment and results.

Study risk of bias was appraised using the Cochrane Risk of Bias 2 (RoB2) tool for randomised controlled trials. The National Institute of health (NIH) National Heart Lung and Blood Institute study quality assessment tool for before-after (pre-post) studies with no control group, was used where intervention studies: 1) had no control group (i.e., single-arm trials) [*n* = 2], or 2) did not measure any of the primary or secondary outcomes of this review [*n* = 2] [36–38]. Two reviewers (SB and TN) independently graded the risk of bias for each study, and any inconsistencies were discussed, and resolved with an additional third reviewer (SF), if required. The RoB2 was individually scored for five domains, as outlined in Figs. 3a, b and 4. To assess the outcome bias domain, we used the most consistently reported outcomes across studies (disease activity and functional ability) [36, 37]. An overall risk of bias was calculated, reflecting a “low risk”, “some concerns” or “high risk” appraisal for each study. In regard to the four studies for which we used the NIH tool, overall risk of bias was assessed by answering 12 questions, and studies were scored as “good”, “fair” or “poor” [38].

Quality of evidence was assessed using Grading of Recommendations Assessment Development and Evaluation (GRADE) analysis, with overall GRADE quality of evidence rated as high, moderate, low or very low quality (Table 2).

### Data synthesis and analysis

For studies that provided suitable data for a meta-analysis, we extracted and collated data into relevant outcomes. Where similar outcomes measures were assessed in different studies, these were grouped appropriately using continuous, inverse variance, random effects models meta-analyses. Where data was not reported by studies, efforts were made to contact authors [*n* = 10] to obtain additional data (i.e., e-mails sent, with follow up 2 weeks later), and if data could still not be obtained, reviewers imputed means and standard deviations [for *n* = 5 interventions], where possible, using the Cochrane Handbook recommended methods [31].

Mean differences (MD) (for outcomes containing studies that used the same measurement scales) and standardised mean differences (SMD) (for outcomes containing studies that used different measurement scales) were tested between experimental groups and control groups (or pre- and post-intervention data, *n* = 2 single-arm studies [8, 39]). As some studies only reported non-normally distributed data for each outcome, normally distributed values were logarithmically transformed to non-normal values, so all studies included in one outcome meta-analysis contained non-normally distributed data [40, 41]. Where this was not possible (for functional ability and depression outcomes), normal and non-normally distributed data were analysed separately. Where interventions used multiple timepoints of assessment, following Cochrane recommendations, we only included the longest timepoint [31]. Also, studies with multiple intervention arms [42, 43] have been merged into 1 entry [31]. We evaluated the 95% confidence intervals (CI) and heterogeneity between studies using the I^2^ statistic, which indicates the variability of the intervention effect due to heterogeneity. A result was considered statistically significant if *p* < 0.05, and interpretation of I^2^ index was made based on Cochrane recommendations, whereby, 0 − 40% = not important; 30 − 60% = moderate heterogeneity; 50 − 90% = substantial heterogeneity; and 75 − 100% = considerable heterogeneity [31]. Review Manager 5.4.1 was used to conduct meta-analyses. Subgroup analysis was conducted to compare the similarity of findings between different types of interventions where ≥ 1 study/timepoint was included in each subgroup. Subgroup analysis focused on 1) target of intervention, i.e., intervention primarily targeting PA or SB, and 2) outcome assessment timepoint, i.e., during/immediately post-intervention or follow-up. Forest plots were generated for each outcome and funnel plots for those meta-analyses that contain ≥ 10 entries.

## Results

### Searching and selection procedure results

**Fig. 1:**
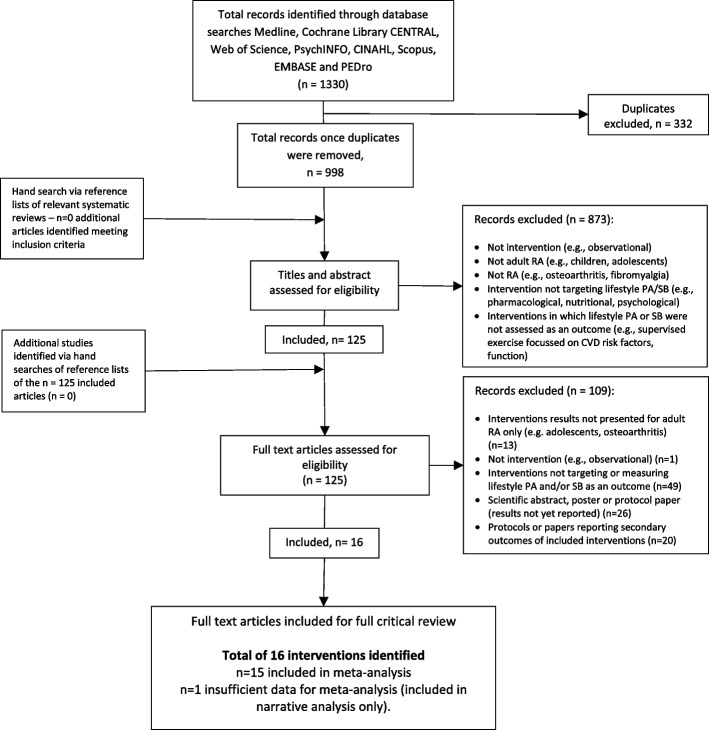
PRISMA diagram of the literature search results. Note: PA= Physical Activity, SB= Sedentary Behaviour, CVD= Cardiovascular Disease, CINAHL= Cumulative Index to Nursing & Allied Health Literature, EMBASE= Excerpta Medica database, PEDro= Physiotherapy Evidence Database, PRISMA= Preferred Reporting Items for Systematic Reviews and Meta-Analyses

The search procedure is described in Fig. 1 (PRISMA flowchart). Initial database searches identified 1330 relevant articles, with a total of 998 articles when duplicates (*n* = 332) were removed. Full texts (*n* = 125) were retained and reviewed against inclusion and exclusion criteria. In total, 15 studies provided sufficient data to be included in this meta-analysis, with two studies providing insufficient information for meta-analysis but is included in narrative analysis [44, 45].

### Characteristics of included studies

This review describes 13 interventions targeting and assessing lifestyle PA only, one intervention with an exclusive focus on SB [21], and two interventions targeting both lifestyle PA and SB [42, 43]. In total, 14 studies were RCTs, and two observational cohort interventions (i.e., single-arm trials, with no control group) [8, 39]. A total of 12 studies were conducted in Europe, two studies in Canada, and two studies in the United States. Intervention duration varied from 5 weeks to 24 months, with an average length of approximately 6 months. Interventions generally included participants with established RA, with only one study recruiting newly diagnosed RA patients [46]. Most participants had low disease activity and few/no severe disabilities. Further characteristics of the included studies can be found in Table 1.

**Table 1:** Summary of findings

***Author, year and country of publication***	***Characteristic:*** *Sample size (n),* *Age (M* ± *SD),* *Gender (% female)*	***IG and CG: design and content***	***Duration & timepoints***	***Assessment of PA and/or SB***	***Disease activity outcome measure and results***	***PA/SB results***	***Secondary Outcome: measure and results***
Brodin et al., 2008 [47] Sweden	IG: 94 54 ± 14.0 72 CG: 134 56 ± 13.9 75	**IG:** Individual coaching program aimed to implement healthy PA. Telephone support given after 1 week, then monthly. 3 monthly function tests to support adherence **CG:** Ordinary physical therapy	1 year baseline PI: 1 year FU: NR	SR: 3 questions- intensity of low, moderate and high intensity PA not validated in RA	DAS28 (ESR): IG: ~ , CG: ~ BGD not assessed	IG: *n* = 26 (34%) ↑, *n* = 19 (20%) ↓ CG: *n* = 23 (20%) ↑, *n* = 31 (23%) ↓ no BGD in number increasing PA	FA (HAQ): IG: ~ , CG: ~ , no BGD QoL (EuroQol VAS): IG: ↑*, CG: ~ , sig. BGD Pain (VAS): IG: ~ , CG: ~ , no BGD
Feldthusen et al., 2016 [48] Sweden	IG: 36 54.2 ± 8.5 88.9 CG: 34 52.7 ± 10.9 88.2	**IG:** Develop self-care plan focussing on tailoring health enhancing PA (reaching adult PA guidelines- i.e., aerobic MPA > 30min, 5d/week; aerobic VPA > 20min, 3d/week; combination of the 2)) and balancing life activities Follow-up support meetings and telephone calls conducted by specialised physical therapists. Frequency of follow-up was individualised. **CG**: Usual care and activities only	12 weeks baseline PI: 12 week FU: 6 months	SR: LTPAI not validated in RA	DAS28 (ESR): IG: ↓ (at post-test and follow-up), CG: ~ , no BGD	LTPAI: IG:↑, CG: ~ sig. BGD between at PI and FU	Fatigue (VAS): IG: ↓*, CG: ↓* at PI and FU, no BGD Pain (VAS): IG: ~ , CG: ~ , no BGD Anxiety (HADS): IG: ↓*, CG: ~ , sig. BGD at PI and FU Depression (HADS): IG: ↓*, CG: ~ , no BGD QoL (EuroQol VAS): IG: ↑*, CG: ~ , sig. BGD at FU
Gilbert et al., 2018 [49] USA	IG: 93 55.0 ± 13.8 82.8 CG: 92 54.7 ± 13.7 84.8	**IG:** Minimum 3-monthly motivational interviews with HCP (in person/telephone)-, individual goal setting, tailored strategies for increasing PA and monitoring progress Progress evaluated in subsequent interviews and further goals set **CG**: Brief PA counselling - physician advice only	24 months baseline DI: 3 months DI: 6 months DI: 12 months PI: 24 months FU: NR	DB: GT1M ActiGraph SR: Yale physical activity scale		Total PA (mins/day): IG: ~ , CG: ~ , no BGD MVPA (mins/day): IG: ~ , CG: ~ , no BGD	FA (HAQ): IG: ~ , CG: ~ , no BGD QoL- Physical (SF-36): IG: ~ , CG: ~ , no BGD QoL- Mental (SF-36): IG: ~ , CG: ~ , sig. BGD at follow-up Pain (HAQ VAS): IG: ~ , CG: ~ , no BGD
Knittle et al., 2015 [50] Netherlands	IG: 38 60.7 ± 11.9 79* CG: 40 64.7 ± 11.5 55*	**IG:** Small group patient education sessions delivered by physical therapist- and one to one motivational interviews and self-regulation coaching FU telephone self-regulation coaching sessions **CG:** Group based patient education session	5 weeks baseline PI: 6 weeks FU: 32 weeks	SR: SQUASH	RADAI: IG: ~ , CG: ~ , sig. BGD at FU in favour of CG	Leisure time PA (mins/week): IG: ↑, CG: ~ , sig. BGD at FU Number active days (days/week): IG: ↑, CG: ~ , sig. BGD at PI and FU	FA (HAQ): IG: ~ , CG: ~ , no BGD Depression (BSI): IG: ~ , CG: ~ , no BGD Fatigue (CIS-20): IG: ~ , CG: ~ , no BGD
Giraudet-Le Quintrec et al., 2007 [25] France	IG: 104 55.3 ± 11.8 86.4 CG: 104 54.3 ± 14.4 85.4	**IG:** multidisciplinary educational intervention- home based exercise prescription and recommendations for leisure PA 8 group weekly face to face, 5-h education program sessions on RA management and physical program, OT, physical therapist, aquatic or relaxation training **CG:** Usual medical care and information booklets with PA recommendations and exercises	12 months baseline DI: 6 months PI: 12 months FU: NR	SR: Baeke questionnaire (assessed leisure time PA (sports + hobbies)) not validated in RA	DAS28: IG: ~ , CG: ~ , no BGD	Leisure PA score: IG: ↓, CG: ↓, no BGD	FA (HAQ): IG: ~ , CG: ~ , no BGD Anxiety (HADS): IG: ~ , CG: ~ , no BGD Depression (HADS): IG: ~ , CG: ~ , no BGD QoL (AIMS2): IG: ~ , CG: ~ , no BGD Fatigue (FACIT-F): IG: ~ , CG: ~ , no BGD
Thomsen et al., 2017 [21] Denmark	IG: 75 59.7 ± 10.7 81 CG: 75 59.5 ± 12.7 80	**IG:** 1: 3 × individual motivational counselling sessions - individual goal setting and self-efficacy, set behavioural goals to reduce sitting, motivation and confidence to encourage behaviour change. Booklets given containing key messages 2: SMS reminders- based on goals (frequency is individualised) **CG:** Current lifestyle	16 weeks baseline PI: 16 weeks- FU: 6 months FU: 22 months	DB: activPAL™ SR: PAS 2.1	DAS28 (CRP): IG: ↓, CG: ↓, no BGD (assessed at FU only)	DB sitting time (hr/day): IG: ↓, CG: ↑, sig. BGD at PI and FU DB standing time (hr/day): IG: ↑, CG: ↓, sig. BGD at PI and FU DB stepping time (hr/day): IG: ↑, CG: ↓, sig. BGD at PI and FU SR sitting at work (hr/day): IG: ↓, CG: ~ , sig. BGD at PI and FU SR sitting in leisure (hr/day): IG: ↓, CG: ↑, sig. BGD at PI and FU	FA (HAQ): IG: ↓*, CG: ↑*, sig. BGD at post-test and follow-up QoL (SF-36): IG: ↑*, CG: ↓*, sig. BGD at post-test and follow-up Pain (VAS): IG: ↓*, CG: ↑*, sig. BGD at post-test and follow-up Fatigue (VAS): IG: ↓*, CG: ↑*, sig. BGD at post-test and follow-up
Van den Berg et al., 2006 [45] Netherlands	IG: 82 49.5 (12.9) median (IQR) 76 CG: 78 49.8 (13.9) median (IQR) 77	**IG:** Internet based PA programme (performed 5x/week)- Individual PA guidance, bicycle ergometer. Participants advised to do other forms of PA as well. Weekly email supervision with physical therapist 3-monthly group meetings – demonstrated new exercises, exchange of experiences. Tailored self-management strategies addressed during meeting **CG:** Internet based general PA training advice	12 months baseline DI: 3 months DI: 6 months DI: 9 months PI: 12 months FU: NR	SR: Questionnaire (number meeting MPA and VPA recommendations) DB: Actilog 3	DAS28 (ESR): IG: ↓, CG: ↓, no BGD	MPA: IG: ↑, CG: ↑, sig. BGD at 6 and 9 months VPA: IG: ↑, CG: ↑, sig. BGD at 6, 9 and 12 months DB PA score: IG: ↓, CG: ↓ (at 6 months), no BGD DB Peak amplitude: IG: ~ , CG: ~ , no BGD DB No. peaks: IG: ~ , CG: ~ , no BGD	FA (HAQ): IG: ↓*, CG: ~ , sig. BGD at 12 months only QoL (RAQoL): IG: ↑*, CG: ↑*, sig. BGD at 9 and 12 months
Veldhuijzen et al., 2021 [51] England	IG: 43 55.4 ± 12.1 63 CG: 45 54.5 ± 13.0 69	**IG:** 3-month exercise program and SDT-based psychological intervention One to one consultations with BC counsellor: to support autonomous motivation for PA RA tailored exercise program: 3 × 30min/wk independent exercise sessions at gym (×2) and home (×1), semi-supervised **CG:** RA tailored exercise program	3 months baseline PI: 3 months FU: 6 months FU: 12 months	SR: IPAQ	DAS28: IG: ~ , CG: ~ , no BGD	MVPA (mins/week): IG: ~ , CG: ↓, sig. BGD at 3, 6 and 12 months	FA (HAQ): IG: ↓*, CG: ↑*, sig. BGD at 6 and 12 months QoL (EQ-5D): IG: ~ , CG: ~ , no BGD Depression (HADS): IG: ~ , CG: ~ , no BGD Anxiety (HADS): IG: ~ , CG: ~ , no BGD Fatigue (MAF): IG: ~ , CG: ~ , no BGD
Li et al., 2020 [43] Canada	IG: 43 54.8 ± 15.4 88.4 CG: 43 55.3 ± 11.5 93	**IG:** 1. in person group education session and individual counselling 2. Wear Fitbit Flex 2 and given PA goals 3. biweekly phone calls from physical therapist trained in motivational interviewing- reviewed PA goals **CG**: Routine activities weeks 1–9, did intervention weeks 10–18 (delay group)	8 weeks baseline PI: Week 9 (post-test IG) PI: Week 18 (post-test CG) FU: Week 27	DB: Sensewear accelerometer		MVPA (mins/day): IG: ↑, CG: ~ , no BGD Purposeful activity (mins): IG: ~ , CG: ~ , no BGD Steps (no./day): IG: ~ , CG: ~ , no BGD Sedentary time (mins): IG: ~ , CG: ~ , no BGD	Depression (PHQ): IG: ~ , CG: ~ , no BGD Pain (MPQ): IG: ↓* (9 weeks), CG: ~ , sig. BGD at 9 weeks Fatigue (FSS): IG: ~ , CG: ~ , no BGD
Katz et al., 2018 [42] USA	PED+: 34 50.2 ± 14.1 88.2 PED: 34 55.9 ± 12.4 88.2 CG: 28 59.1 ± 12.5* 85.7	**IG:** 1. PED+: individualized step-count goals + pedometer + step-monitoring diary: booklet and discussion, pedometer, step diary and individualised daily step targets. Follow-up- target review phone call every 2 weeks 2. PED: pedometer + diary, NO targets: booklet and discussion, pedometer and diary to record daily pedometer steps. Follow-up- step count recorded via phone call every 2 weeks **CG:** education booklet and discussion on PA benefits	21 weeks baseline DI: 10 weeks PI: 21 weeks FU: NR	DB: Jawbone pedometer DB: Fitbit	RADAI (1–10): PED+: ↓, PED: ↓, CG: ↑ (at week 21), sig. BGD (lower in PED and PED+ than CG)	Steps (no./day): PED+: ↑, PED: ↑, CG: ~ , sig. BGD (changes within PED and PED+ differed from CG) % sedentary participants: PED+: ↓, PED: ↓, CG: ↑, sig. BGD % achieving healthy PA: PED+: ↑, PED: ↑, CG: ~ , no BGD	FA (HAQ): PED+: ↓*, PED: ↓*, CG: ~ , sig. BGD in PED+ vs CG at 21 weeks Pain (PROMIS): PED+: ↓*, PED: ↓*, CG: ~ , no BGD Fatigue (PROMIS): PED+: ↓*, PED: ↓*. CG: ~ , no BGD Depression (PHQ): PED+: ↓*, PED: ↓*, CG: ~ , no BGD
Nordgren et al., 2015 [8] Sweden	IG: 220 59 ± 8.8 81	**IG:** Health enhancing PA** (**HEPA) programme 1. 30+ mins MPA on most days- given pedometer and access to webpage for step registration to encourage daily PA 2. 2 × circuit training sessions/week in gym 3. biweekly support group meetings by PTs Alternative types of HEPA were encouraged- competitions, monitor aerobic capacity, weekly texts Expert lectures **CG:** No control, single-arm trial	2 years baseline DI: 12 months FU: NR	SR: IPAQ-SF SR: modified ESAI		% meeting current HEPA: IG: ↑ (at 1 year), ↓ from year 1 to year 2 (82% to 75%) % maintained (> 6 months) HEPA: IG: ↑ 0 to 37% (at 1 year), ↓ from year 1 to year 2 (841% to 27%)	FA (HAQ): IG: ↓* QoL (EQ-5D): IG: ↑* Pain (VAS): IG: ↓* Fatigue (VAS): IG: ~
Lange et al., 2020 [52] Sweden	IG: 24 73.5 ± 2.7 75.0 CG: 23 74.0 ± 2.1 78.3	**IG:** Moderate-high intensity, aerobic and resistance exercise with person-centred guidance 3 sessions/week tailored gym based exercise: semi-supervised. Home based exercise: LPA 5 days/week and home exercises 2x/week Telephone support 7 months post intervention **CG:** Encouraged to perform home-based light intensity exercise	20 weeks baseline FU: 4 years	SR: LTPAI SR: ESAI	DAS28 (ESR): IG: ~ , CG: ↑, sig. BGD	LTPAI: IG: ↑, CG: ~ , no BGD ESAI- current HEPA: IG: 33%, CG: 26%, no BGD ESAI- maintained HEPA: IG: 25%, CG: 17%, no BGD	FA (HAQ)I: IG: ~ , CG: ~ , no BGD QoL (VAS): IG: ~ , CG: ↓*, sig. BGD Pain (VAS): IG: ~ , CG: ↑*, no BGD Fatigue (VAS): IG: ~ , CG: ↑*, no BGD
John et al., 2013 [44] England	IG: 52 62.2 ± 10.6 71 CG: 58 60.8 ± 10.7 74	**IG:** Cognitive behavioural education intervention 3 × interactive small group meetings by HCPs The important role of lifestyle modifications discussed, and individuals challenged to (using probing behavioural techniques), and commit to, a specific behaviour change Weekly progress reviews encouraged to self-monitor **CG:** Information leaflet	8 weeks baseline PI: 8 weeks FU: 6 months	SR: IPAQ		MET PA (mins/week): no BGD (WGD not assessed)	
Garner et al., 2018 [46] Canada	IG: 14 45 ± 10 93 CG: 14 49 ± 14 71	**IG:** Individualised counselling intervention on PA and dietary intake 3 × individualized visits to review strategies on: 1. Nutrition: with dietician, food questionnaire, reviewed diet recommendations, asked questions about diet. 2. PA: with rheumatology PT. Reviewed current PA and fitness tests results, instructions on PA guidelines, exercises to improve fitness. **CG:** Standard care	6 months baseline PI: 6 months FU: NR	DB: Pedometer	DAS28: IG: ↓, CG: ↓, no BGD	Steps (no./week): IG: ↑ +9,583 steps, CG: ↑ +6,696 steps, no BGD	FA (HAQ): no within group data reported, no BGD
Cramp et al., 2020 [39] England	IG: 12 58 (range: 23–79) 75	**IG:** 4 × group sessions: set goals, autonomy support, facilitate relatedness, group discussion, action plans tailored, individualised, to promote intrinsic motivation, peer support, self-monitoring (daily diaries and pedometers to take home) incorporated to promote self-efficacy and BC. One to one session: individual support to meet specific needs- discussion of individual PA barriers, strategies to overcome these **CG**: No control, single-arm trial	12 weeks baseline PI: 12 weeks FU: NR	SR: IPAQ-SF		IPAQ PA: IG: ~ (1 = ↑, 1 = ↓)	FA (modified HAQ): IG: 3 = ↑ QoL (EQ-5D): IG: ~ Pain (VAS): IG: 4 = ↑ Fatigue (BRAF-NRS): IG: 6 = ↑ (better), 3 = ↓ (worse), 2 = ~ didn’t test for significance
McKenna et al., 2021 [53] Ireland	IG: 10 58 ± 7.4 100 CG: 10 56 ± 7.9 100	**IG:** Walking based exercise intervention based on ACSM, WHO and EULAR guidelines Sessions increased in length, intensity and duration each week from 2 to 5 sessions by week 8. Incrementally longer walks and more challenging targets. Progress self-monitored. Unsupervised sessions performed at time and location of choice **CG:** verbal and written instructions about benefits of exercise in RA	8 weeks baseline PI: Week 9 FU: NR	DB: activPAL™	CDAI: IG: ↓ (-0.7), CG: ↑ (+0.7) (didn’t test for significance, BGD not assessed)	MVPA (mins/day): IG: ↑, CG: ~ (BGD not assessed)	FA (HAQ): IG:↓ (-0.6), CG: ↑ (+0.14) QoL (VAS): IG: ↑ (+10.4), CG: ↑ (+0.3) Pain (VAS): IG: ↓, CG: ~ Fatigue (BRAF-NRS): IG: ↓ (-11), CG: ↑ (+1) (didn’t test for significance, BGD not assessed)

*WGD* Within group difference, *BGD* Between group difference, ~  = no significant change, ↑ = increase (not significant), ↓ = decrease (not significant), ↑* = increase (significant), ↓* = decrease (significant)

*USA* United States of America, *M* ± *SD* Mean ± standard deviation, *IG* Intervention group, *CG* Control group, *DI* During intervention, *PI* Post-intervention, *FU* Follow-up, *NR* Not reported, *DB* Device-based, *SR* Self-report, *PA* Physical activity, *MVPA* Moderate-to-vigorous physical activity, *MPA* Moderate physical activity, *VPA* Vigorous physical activity, *LTPAI* Leisure time PA index, *PAS 2.1* Physical Activity Scale 2.1, *HEPA* Health enhancing physical activity, *SQUASH* Short Questionnaire to Assess Health-Enhancing Physical Activity, *IPAQ-SF* International Physical Activity Questionnaire- short form, *ESAI* Exercise Stage Assessment Instrument, *DAS28* Disease activity score- 28, *ESR* Erythrocyte sedimentation rate, *CRP* C-reactive protein, *CDAI* Clinical disease activity index, *RADAI* Rheumatoid Arthritis Disease Activity Index, *FA* Functional ability, *HAQ* Health assessment questionnaire, *QoL* Quality of life, *VAS* Visual analogue scale, *HADS* Hospital Anxiety and Depression Scale, *SF-36* Short Form-36, *BSI* Brief Symptom Inventory, *CIS-20* Checklist of Individual Strengths, *AIMS2* Arthritis Impact Measurement Scale, *FACIT-F* Functional Assessment of Chronic Illness Therapy – Fatigue scale, *RAQoL* Rheumatoid Arthritis Quality of Life questionnaire, *EQ-5D* EuroQol-5 dimensions questionnaire, *MAF* Multidimensional Assessment of Fatigue, *PHQ* Patient health questionnaire, *FSS* Fatigue severity scale, *MPQ* McGill pain questionnaire, *PROMIS* Patient-Reported Outcomes Measurement Information System, *BRAF-NRS* Bristol Rheumatoid Arthritis Fatigue—Numerical Rating Scales

### Effect of interventions

#### Primary outcome

Measurement tools and intervention results regarding disease activity are reported in Table 1. In brief, disease activity was reported by 11 studies, with some heterogeneity in the measurement tools. In total, eight studies used the disease activity score 28 (DAS28) [54], two used the Rheumatoid Arthritis Disease Activity Index (RADAI) [55], and one used the Clinical Disease Activity Index (CDAI) [56]. All measures of disease activity were based on patient or clinician physical assessment, with only the DAS28 having a serological marker of inflammation included as an objective element.

**Fig. 2:**
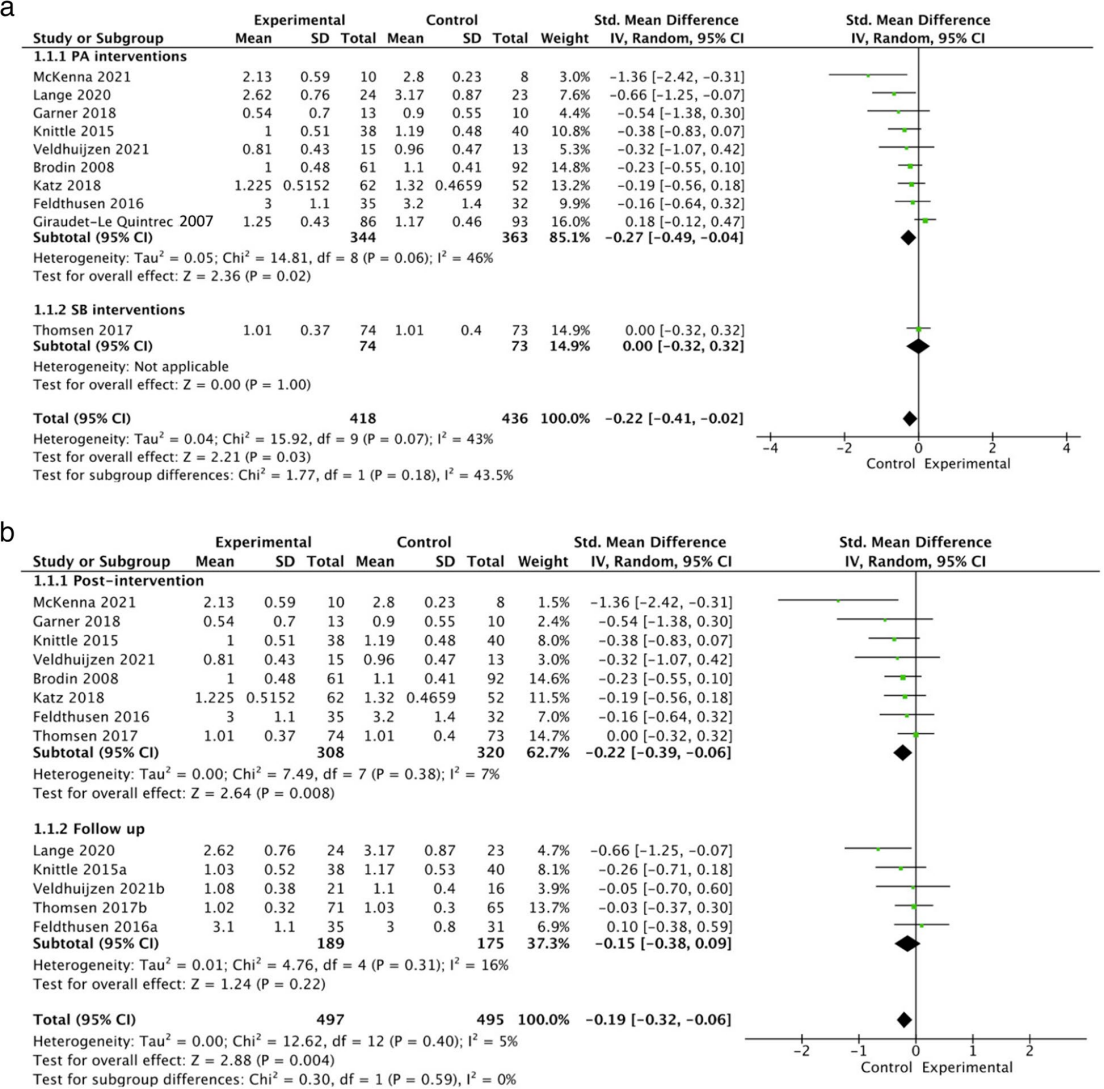
**a** The effects of interventions on disease activity with physical activity vs sedentary behaviour intervention subgroup analysis. **b** The effects of interventions on disease activity with post-intervention vs follow-up subgroup analysis. Note: Where studies reported data from multiple post-intervention timepoints, these were included as separate studies in each meta-analysis (e.g., Thomsen 2017 = 16-week timepoint, Thomsen 2017a = 10-month timepoint). Where studies reported data from multiple interventional arms, these were included as separate studies in each meta-analysis (e.g., Katz 2018a = PED intervention group, Katz 2018a+ = PED+ intervention group).SD = standard deviation, 95% CI = 95% confidence interval

The meta-analysis included data from 10 studies, comprising *n* = 854 participants (*n* = 418 in intervention groups, *n* = 436 in control groups). Results showed a statistically significant positive effect of lifestyle PA and SB interventions on reducing disease activity compared to the control group, with SMD of -0.22 (95% CI -0.41 to -0.02, I^2^ = 43%, z = 2.21, *p* = 0.03) (Fig. 2a, Supplementary Fig. 27). GRADE analysis (Table 2) revealed results were not affected by the inclusion of studies with varied risk of bias, with moderate quality of evidence for this outcome due to studies being varied in their primary aims.

**Table 2:** GRADE analysis for disease activity and secondary outcomes

***Summary of findings table according to GRADE analysis***							***Evaluation components to lower quality***						***Evaluation components to higher quality***		
***Outcome***	***Intervention Effects (SMD/MD)***	***No. studies***	***No. Participants IG***	***No. Participants CG***	***GRADE***	***Comments***	***Methodological design start point***	***Risk of bias***	***Inconsistency of results***	***Indirectness***	***Imprecision***	***Publication bias***	***Large effect***	***Dose response***	***Confounding***
Disease Activity	SMD = -0.22 [-0.41, -0.02]	10	418	436	Moderate ⨁⨁⨁◯	We are moderately confident in the effect estimate: The true effect is likely to be close to the estimate of the effect, but there is a possibility that it is substantially different	Mixture of RCTs and non-RCTs: High quality	60% studies had moderate RoB, 40% had high RoB: no downgrade	Even though we used a random effect model meta-analysis, we consider heterogeneity as an index of inconsistency. I2 = 43%, not considerable (< 75%), no downgrade	Very few studies with disease activity as primary aim. Downgrade 1 level	*N* = 854 sample size, very large so unlikely to be imprecise. No downgrade	We used an exhaustive searching approach (i.e. scientific databases, grey literature, scientific organizations). Funnel plot is asymmetrical, downgrade 1 level	z score = 2.21, large effect. Upgrade 1 level	N/A	We found no confounding factors that indicate upgrading
Functional Ability (normal)	MD = -0.21 [-0.37, -0.06]	8	482	491	Very Low ⨁◯◯◯	Our confidence in the effect estimate is limited: The true effect may be substantially different from the estimate of the effect.	Mixture of RCTs, non-RCTs and observational cohort studies: Moderate quality	50% studies had moderate RoB, 50% had high RoB: downgrade 1 level	Even though we used a random effect model meta-analysis, we consider heterogeneity as an index of inconsistency. I2 = 85%, considerable heterogeneity, downgrade 1 level	Studies highly varied in primary aim, with very few with function as primary aim. Downgrade 1 level	*n* = 973, unlikely to be imprecise, no downgrade	We used an exhaustive searching approach (i.e. scientific databases, grey literature, scientific organizations). No major bias in the funnel plots. No downgrade	z score = 2.66, large effect. Upgrade 1 level	N/A	We found no confounding factors that indicate upgrading
Functional Ability (non-normal)	MD = -0.00 [-0.06, 0.06]	4	209	223	High ⨁⨁⨁⨁	We are very confident that the true effect lies close to that of the estimate of the effect.	Mixture of RCTs and non-RCTs: High quality	100% studies had moderate RoB: no downgrade	Even though we used a random effect model meta-analysis, we consider heterogeneity as an index of inconsistency. I2 = 2%, no heterogeneity	most studies primary aim is function, No downgrade	*n* = 432, unlikely to be imprecise, no downgrade	We used an exhaustive searching approach (i.e. scientific databases, grey literature, scientific organizations). No major bias in the funnel plots. No downgrade	z score = 0.06, no effect. No upgrade	N/A	We found no confounding factors that indicate upgrading
Pain	SMD = -0.13 [-0.79, 0.53]	10	586	640	Very Low ⨁◯◯◯	We have very little confidence in the effect estimate: The true effect is likely to be substantially different from the estimate of effect	Mixture of RCTs, non-RCTs and observational cohort studies: Moderate quality	70% studies moderate, 30% high RoB: no downgrade	Even though we used a random effect model meta-analysis, we consider heterogeneity as an index of inconsistency. I2 = 96%, considerable heterogeneity, downgrade 1 level	Studies varied in primary aim, with 6 with pain as primary aim (< 50%). Downgrade 1 level	*N* = 1226, unlikely to be imprecise, no downgrade	We used an exhaustive searching approach (i.e. scientific databases, grey literature, scientific organizations). Funnel plot is asymmetrical, downgrade 1 level	z = 0.4, no effect. No upgrade	N/A	We found no confounding factors that indicate upgrading
Fatigue	SMD = -0.42 [-0.63, -0.21]	10	534	523	Moderate ⨁⨁⨁◯	We are moderately confident in the effect estimate: The true effect is likely to be close to the estimate of the effect, but there is a possibility that it is substantially different	Mixture of RCTs, non-RCTs and observational cohort studies: Moderate quality	60% moderate, 40% high RoB: no downgrade	Even though we used a random effect model meta-analysis, we consider heterogeneity as an index of inconsistency. I2 = 56%, no downgrade	studies had varied primary aims, downgrade 1 level	*n* = 1057, unlikely to be imprecise, no downgrade	We used an exhaustive searching approach (i.e. scientific databases, grey literature, scientific organizations). No major bias in the funnel plots. No downgrade	z = 3.87, large effect. Upgrade 1 level	N/A	We found no confounding factors that indicate upgrading
Anxiety	SMD = -0.19 [-0.95, 0.57]	3	82	70	Very Low ⨁◯◯◯	We are moderately confident in the effect estimate: The true effect is likely to be close to the estimate of the effect, but there is a possibility that it is substantially different	Mixture of RCTs and non-RCTs: High quality	66% moderate, 33% high risk: no downgrade	Even though we used a random effect model meta-analysis, we consider heterogeneity as an index of inconsistency. I2 = 81%, considerable heterogeneity, downgrade 1 level	< 50% studies had anxiety in their primary aim, downgrade 1 level	*n* = 152, small sample so likely to be imprecise, downgrade 1 level	We used an exhaustive searching approach (i.e. scientific databases, grey literature, scientific organizations). No major bias in the funnel plots. No downgrade	z = 0.48, little effect. No upgrade	N/A	We found no confounding factors that indicate upgrading
Depression (non-normal)	MD = -0.92 [-2.71, 0.87]	2	62	54	Low ⨁⨁◯◯	We are moderately confident in the effect estimate: The true effect is likely to be close to the estimate of the effect, but there is a possibility that it is substantially different	Mixture of RCTs and non-RCTs: High quality	100% studies had moderate RoB: no downgrade	Even though we used a random effect model meta-analysis, we consider heterogeneity as an index of inconsistency. I2 = 49%, no downgrade	< 50% studies had depression in their primary aim, downgrade 1 level	*n* = 116, small sample so likely to be imprecise, downgrade 1 level	We used an exhaustive searching approach (i.e. scientific databases, grey literature, scientific organizations). No major bias in the funnel plots. No downgrade	z = 1.01,, little effect. No upgrade	N/A	We found no confounding factors that indicate upgrading
Depression (normal)	SMD = -0.39 [-1.08, 0.30]	4	119	115	Very Low ⨁◯◯◯	We are moderately confident in the effect estimate: The true effect is likely to be close to the estimate of the effect, but there is a possibility that it is substantially different	Mixture of RCTs and non-RCTs: High quality	25% studies had moderate Rob, 75% high risk: downgrade 1 level	Even though we used a random effect model meta-analysis, we consider heterogeneity as an index of inconsistency. I2 = 83%, considerable heterogeneity, downgrade 1 level	< 50% studies had depression in their primary aim, downgrade 1 level	*n* = 234, small sample so likely to be imprecise, downgrade 1 level	We used an exhaustive searching approach (i.e. scientific databases, grey literature, scientific organizations). No major bias in the funnel plots. No downgrade	z = 1.11, little effect. No upgrade	N/A	We found no confounding factors that indicate upgrading
Quality of Life	SMD = 0.29 [-0.05, 0.62]	9	685	738	Very Low ⨁◯◯◯	We have very little confidence in the effect estimate: The true effect is likely to be substantially different from the estimate of effect	Mixture of RCTs, non-RCTs and observational cohort studies: Moderate quality	92% studies had moderate RoB, 8% high risk: no downgrade	Even though we used a random effect model meta-analysis, we consider heterogeneity as an index of inconsistency. I2 = 87%, considerable heterogeneity, downgrade 1 level	< 50% studies had QoL in their primary aim, downgrade 1 level	*n* = 1423, unlikely to be imprecise, no downgrade	We used an exhaustive searching approach (i.e. scientific databases, grey literature, scientific organizations). Funnel plot is asymmetrical, downgrade 1 level	z = 1.69, little effect. No upgrade	N/A	We found no confounding factors that indicate upgrading
Sedentary Time	MD = -46.80 [-162.30, 68.70]	2	128	130	Very Low ⨁◯◯◯	We are very confident that the true effect lies close to that of the estimate of the effect.	Mixture of RCTs and non-RCTs: High quality	50% studies had moderate RoB, 50% high risk: downgrade 1 level	Even though we used a random effect model meta-analysis, we consider heterogeneity as an index of inconsistency. I2 = 88% considerable heterogeneity, downgrade 1 level	> 50% studies primary aim was to target SB, no downgrade	*n* = 258, small sample so likely to be imprecise, downgrade 1 level	We used an exhaustive searching approach (i.e. scientific databases, grey literature, scientific organizations). No major bias in the funnel plots. No downgrade	z = 0.79, no effect. No upgrade	N/A	We found no confounding factors that indicate upgrading
Steps	SMD = 0.30 [0.03, 0.57]	3	130	95	Moderate ⨁⨁⨁◯	We are very confident that the true effect lies close to that of the estimate of the effect.	Mixture of RCTs and non-RCTs: High quality	33% studies had moderate RoB, 67% high risk: downgrade 1 level	Even though we used a random effect model meta-analysis, we consider heterogeneity as an index of inconsistency. I2 = 0%, no heterogeneity, no downgrade	All studies outcomes were some form of PA/SB measure so these are sufficiently similar, no downgrade	*n* = 225, small sample so likely to be imprecise, downgrade 1 level	We used an exhaustive searching approach (i.e. scientific databases, grey literature, scientific organizations). No major bias in the funnel plots. No downgrade	z = 2.15, large effect. Upgrade 1 level	N/A	We found no confounding factors that indicate upgrading
MVPA	SMD = 1.21 [-0.01, 2.44]	7	307	292	Moderate ⨁⨁⨁◯	We are very confident that the true effect lies close to that of the estimate of the effect.	Mixture of RCTs and non-RCTs: High quality	65% studies moderate risk, 35% high risk: no downgrade	Even though we used a random effect model meta-analysis, we consider heterogeneity as an index of inconsistency. I2 = 98%, considerable heterogeneity, downgrade 1 level	> 50% studies primary aim was to target MVPA, no downgrade	*n* = 599, unlikely to be imprecise, no downgrade	We used an exhaustive searching approach (i.e. scientific databases, grey literature, scientific organizations). No major bias in the funnel plots. No downgrade	z = 1.94, little effect. No upgrade	N/A	We found no confounding factors that indicate upgrading
Total PA	SMD = 0.03 [-0.37, 0.43]	4	200	189	Moderate ⨁⨁⨁◯	We are moderately confident in the effect estimate: The true effect is likely to be close to the estimate of the effect, but there is a possibility that it is substantially different	Mixture of RCTs and non-RCTs: High quality	50% had high RoB: downgrade 1 level	Even though we used a random effect model meta-analysis, we consider heterogeneity as an index of inconsistency. I2 = 71%, no heterogeneity, no downgrade	> 50% studies primary aim was to target PA, no downgrade	*n* = 389, unlikely to be imprecise, no downgrade	We used an exhaustive searching approach (i.e. scientific databases, grey literature, scientific organizations). No major bias in the funnel plots. No downgrade	z = 0.13, no effect. No upgrade	N/A	We found no confounding factors that indicate upgrading
Light/leisure PA	SMD = 0.45 [0.27, 0.64]	4	238	225	High ⨁⨁⨁⨁	We are very confident that the true effect lies close to that of the estimate of the effect.	Mixture of RCTs and non-RCTs: High quality	75% studies had moderate RoB, 25% high risk: no downgrade	Even though we used a random effect model meta-analysis, we consider heterogeneity as an index of inconsistency. I2 = 0%, no heterogeneity	> 50% studies primary aim was to target some form of PA or SB, no downgrade	*n* = 463, unlikely to be imprecise, no downgrade	We used an exhaustive searching approach (i.e. scientific databases, grey literature, scientific organizations). No major bias in the funnel plots. No downgrade	z = 4.79, large effect. Upgrade 1 level	N/A	We found no confounding factors that indicate upgrading

An overall quality score is obtained using the assessments of risk of bias, inconsistency, indirectness, imprecision, publication bias, large effect, dose response and confounding factors for all outcomes

*IG* Intervention group, *CG* Control group, *FA* Functional ability, *MVPA* Moderate to vigorous physical activity, *PA* Physical activity, *SB* Sedentary behaviour, *SMD* Standardised mean difference, *MD* Mean difference, *RCT* Randomised controlled trial, *RoB* Risk of bias

##### Subgroup analysis

Subgroup analyses showed that only lifestyle PA interventions, but not the single SB intervention, had statistically significant effects on disease activity (Fig. 2a). PA interventions (*n* = 9707 participants) demonstrated an SMD of -0.27 (95% CI -0.49 to -0.04, I^2^ = 46%, z = 2.36, *p* = 0.02), whilst the SB intervention (*n* = 1147 participants) displayed an SMD of 0.00 (95% CI -0.32 to 0.32, z = 0.00, *p* = 1.0), however, no differences between groups were detected (*p* > 0.05). When examining changes relative to different assessment timepoints, whilst lifestyle PA interventions showed statistically significant during or immediately post-intervention effects on disease activity, no intervention effects were demonstrated at follow-up (Fig. 2b, Supplementary Fig. 28). It was not possible to perform this subgroup analysis on SB interventions due to insufficient data.

#### Secondary outcomes

##### Lifestyle PA and SB

In total, 11 studies employed self-report methods to assess lifestyle PA and SB outcomes (sedentary time, steps, MVPA, total PA and leisure/light intensity PA), and seven studies used device-based assessments (i.e., pedometers [42, 46] and accelerometers [21, 43, 45, 49, 53]). Only two interventions used both self-report and device-based measures [21, 45].

Meta-analysis results revealed statistically significant intervention effects on daily steps with an SMD of 0.30 (95% CI 0.03 to 0.57, I^2^ = 0%, z = 2.15, *p* = 0.03) and leisure/light intensity PA with an SMD of 0.45 (95% CI 0.27 to 0.64, I^2^ = 0%, z = 4.79, *p* < 0.001), with nearing statistically significant intervention effects reported for MVPA (SMD = 1.21 (95% CI -0.01 to 2.44, I^2^ = 98%, z = 1.94, *p* = 0.05) (Supplementary Figs. 9–13). Subgroup analysis revealed statistically significant PA intervention effects on steps and leisure/light intensity PA, and SB intervention effects on leisure/light intensity PA (Supplementary Figs. 9–13). Further estimation of subgroup differences between PA vs SB interventions were not possible due to the limited number of studies included in meta-analyses. Post-intervention effects were demonstrated for steps and leisure/light intensity PA, with statistically significant follow-up effects displayed for leisure/light intensity PA only (Supplementary Figs. 22–26, 35). Method of assessment demonstrated little effect on intervention success at changing lifestyle PA or SB, however interventions targeting a particular dimension or domain of lifestyle PA, such as steps, MVPA and leisure/light intensity PA, were more effective than those targeting total PA or sedentary time.

##### Patient- and clinician-important outcomes

Meta-analyses reported statistically significant intervention effects on: measures of functional ability (normally distributed) with MD of -0.21 (95% CI -0.37 to -0.08, I^2^ = 85%, z = 2.66, *p* < 0.01) and fatigue with a SMD of -0.42 (95% CI -0.63 to -0.21, I^2^ = 56%, z = 3.87 *p* < 0.001). These effects all demonstrated improvements in outcomes. No other statistically significant results were observed for patient- and clinician-important health outcomes (Supplementary Figs. 1–8 and 29–34). Subgroup analysis showed statistically significant lifestyle PA intervention effects on increasing functional ability (normally distributed data) and decreasing fatigue. In addition, there were statistically significant SB intervention effects on increasing functional ability (normally distributed data), decreasing pain and fatigue, and increasing quality of life (Supplementary Figs. 1–8). Furthermore, immediate positive post-intervention effects were seen for functional ability (normally distributed data) and fatigue, whilst effects at follow-up were demonstrated for reducing pain and improving quality of life (Supplementary Figs. 14–21 and 30–34).

##### Changes in lifestyle PA and SB in the context of patient- and clinician-important outcomes

Of the two studies demonstrating statistically significant between- and within-group improvements in disease activity, both also displayed increases in intervention group leisure/light intensity PA [42, 52]. All studies reporting functional ability improvements also displayed intervention effects for lifestyle PA and/or SB [8, 21, 42, 45, 51]. Of the four studies reporting reductions in pain [8, 21, 42, 43], three also reported statistically significant reductions in SB, and increased steps and leisure/light intensity PA [8, 21, 42]. For fatigue, two of the three studies demonstrating reductions in fatigue post-intervention also observed statistically significant decreases in SB, and increases in steps and leisure/light intensity PA [21, 42]. Finally, four of the seven studies reporting improvements in mental health, psychological wellbeing or quality of life following intervention, also demonstrated significantly increased lifestyle PA and/or reduced SB [8, 21, 45, 48].

### Risk of bias assessment results

**Fig. 3:**
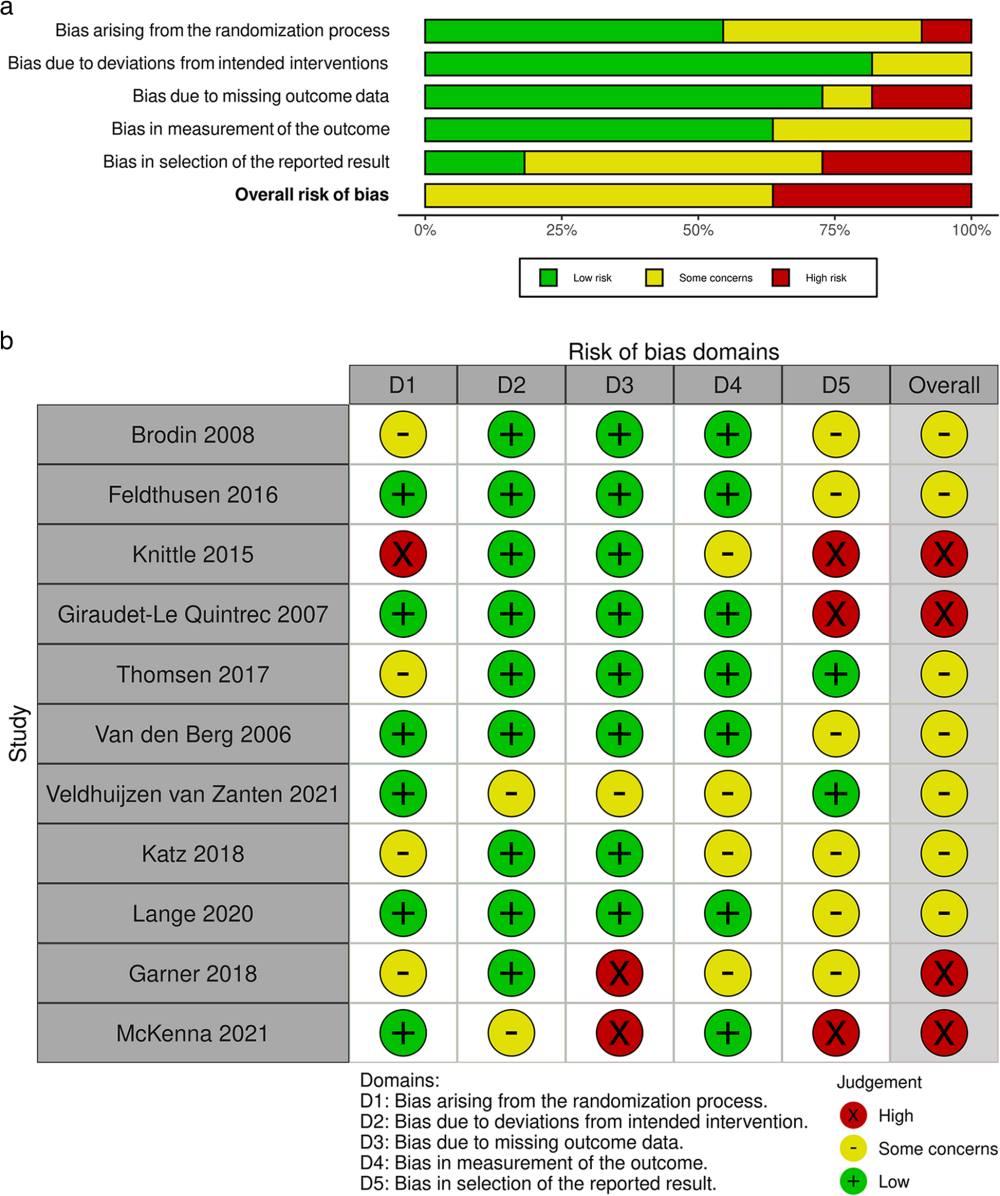
**a** Summary Risk of bias assessment for Disease Activity. Note: ROB domains include; (1) Bias arising from the randomization process; (2) Bias due to deviations from intended interventions; (3) Bias due to missing outcome data; (4) Bias in measurement of the outcome; and (5) Bias in selection of the reported result. **b** Risk of bias assessment for Disease Activity. Note: With disease activity as the outcome of interest: 55% studies showed low risk of bias, 36% showed some concerns and 9% had high risk of bias arising from the randomisation process, due to insufficient information about blinding in the randomisation process. In “deviations from intended interventions”, 82% studies displayed low risk of bias, and only 18% had some concerns, indicating that few studies appeared to deviate from their protocol or methods.73% included studies demonstrated low risk, 9% had some concerns and 18% had high risk of bias due to missing outcome data, as some studies were feasibility studies, with small sample sizes. For the “bias in measurement of the outcome” domain, 55% studies demonstrated low risk and the remaining 45% displayed some concerns. This domain was mostly low risk due to the disease activity measures being valid and partially objective in nature. For “bias in selection of the reported result”, 18% studies showed low risk, with 55% showing some concerns and 27% with high risk of bias, due to missing data at some pre-specified timepoints

**Fig. 4:**
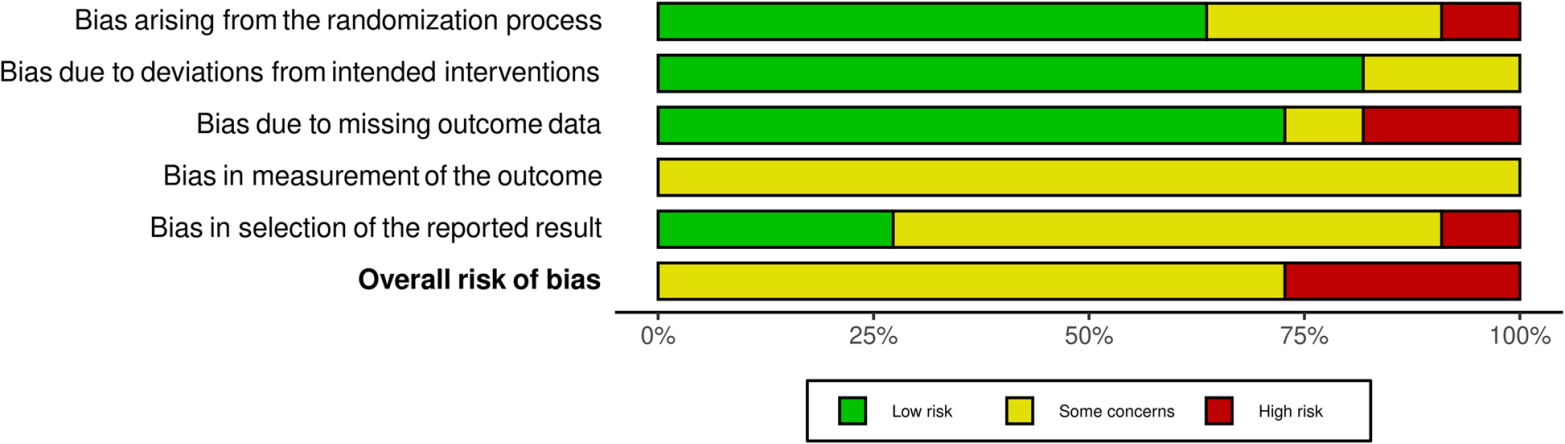
Summary Risk of bias assessment for Functional Ability. Note: ROB domains include; (1) Bias arising from the randomization process; (2) Bias due to deviations from intended interventions; (3) Bias due to missing outcome data; (4) Bias in measurement of the outcome; and (5) Bias in selection of the reported result

A summary of the RoB2 assessment with disease activity and functional ability as outcomes is illustrated in Figs. 3a and 4, respectively. To summarise, of the 11 studies that used disease activity as an outcome, none displayed a low risk of bias, seven displayed some concerns [21, 42, 45, 47, 48, 51, 52], and four high risk of bias [25, 46, 50, 53]. For the 11 studies with a functional ability outcome, no studies were low risk, eight showed some concerns [21, 25, 42, 45, 47, 49, 51, 52], and three high risk of bias [46, 50, 53]. Full domain results of RoB2 analysis for disease activity can be visualised in Fig. 3b.

## Discussion

This systematic review with meta-analysis identified 16 lifestyle PA and SB interventions in RA patients, and aimed to evaluate their effect on disease activity, lifestyle PA and SB, and OMERACT patient- and clinician-important outcomes in people with RA.

### Overview of main outcomes

Lifestyle PA interventions demonstrated statistically significant effects on reducing disease activity in individuals with RA. Statistically significant effects were also observed for steps, and leisure/light intensity PA. The majority of interventions which displayed statistically significant increases in PA and/or reductions in SB also revealed improvements in patient- and clinician-important outcomes. Specifically, lifestyle PA interventions were effective at improving functional ability and fatigue, and the one SB intervention reported statistically significant effects on all secondary outcomes assessed in their study (functional ability, pain, fatigue, quality of life). Despite this, findings also revealed lifestyle PA and SB interventions were unsuccessful at targeting sedentary time, total PA, anxiety and depression in people with RA, although close to statistically significant effects were visualised for MVPA (Supplementary Fig. 11). Together this suggests lifestyle PA and SB interventions may be more effective at increasing specific domains and dimensions of PA, and improve specific health outcomes more so than other outcomes in people with RA.

### Completeness and applicability of evidence

Our analysis showed that lifestyle PA interventions may be beneficial to treat disease activity in RA, supporting findings from observational studies [7, 57]. Those interventions demonstrating efficacy in improving disease activity displayed similar characteristics: longer in duration (approximately 20 weeks), with a primary focus on promoting light-to-moderate intensity PA or walking [42, 52]. These results add to emerging evidence which suggests that light-intensity PA is linked with disease activity and inflammation in people with RA [13, 58]. Together, a longer length of intervention which targets light-to-moderate intensity PA may be required for detectable changes in disease activity in people with RA. The one SB intervention conducted in people with RA showed no effects on disease activity. Further experimental studies investigating the role of SB for disease activity, and SB interventions in people with RA are needed to confirm these findings.

We provide evidence that lifestyle PA and SB interventions are effective at increasing leisure/light intensity PA and daily steps in people with RA. Interventions demonstrated a reduction in sedentary time by 47 min/day. O’Brien, Ntoumanis [59] previously found a reduction in sedentary time by 33 min/day was sufficient to display clinically significant reductions in pain and fatigue. Fenton, Veldhuijzen Van Zanten [58] also revealed that reducing sedentary time by 68 min/day equated to a significant 5.5% reduction in cardiovascular disease risk. Together, this suggests that although our results of a 47 min/day reduction in sedentary time resulting from lifestyle PA and SB interventions did not reach statistical significance, findings are clinically significant.

It is interesting that previous research in non-RA populations has reported that interventions exclusively targeting SB are more effective than PA-only or combined PA + SB interventions, when aiming to reduce sedentary time [60]. Our meta-analysis reported similar findings, but included only one SB intervention, limiting our ability to conduct sufficiently powered analyses and draw firm conclusions in the case of RA. Further SB interventions are therefore needed to elucidate if targeting and reducing SB offers an avenue for interventions to improve disease activity and other core patient- and clinician-important outcomes in people with RA. The intervention that exclusively targeted SB in this review, demonstrated statistically significant reductions in sitting time, alongside increases in standing and stepping time and improvements in RA outcomes [21], suggesting there is value in interventions targeting SB in this patient group.

Lifestyle PA and SB interventions may play a role in improving OMERACT patient- and clinician-important outcomes. Our findings agree with results of previous systematic and narrative reviews highlighting the effects of general PA and exercise training on health outcomes, in people living with RA [7, 57, 61]. In terms of the clinical relevance, previously two studies found the minimal clinically important difference (MCID) (i.e., the smallest change in an outcome that can be perceived as clinically meaningful) of the Hospital Anxiety and Depression Scale (HADS) was 1.5 and 0.5–5.6 in patients with chronic obstructive pulmonary disease and cardiovascular disease, respectively [62, 63]. For the Stanford Health Assessment Questionnaire (HAQ), Bruce and Fries [64] previously demonstrated an MCID of 0.10–0.22 in RA patients. Our finding of a reduction of 0.92 and 0.21 in respect to the HADS (depressive symptoms subscale) and HAQ respectively, may therefore be clinically relevant for people with RA. As such, results suggest that these patients may achieve tangible mental and physical health benefits from lifestyle PA and/or SB interventions.

The lack of beneficial effect of interventions on some secondary outcomes may be due to heterogeneity between the interventions, in terms of intervention length, content and method of outcome assessment. This was indicated by large I^2^ statistic for these outcomes (I^2^ = 0–98%). This highlights the need for a consensus on optimal measurement methods and reporting for these health outcomes (e.g., MD, rather than SMD), in order for interventions effects on outcomes to be reliably and accurately assessed in future meta-analyses.

Findings from subgroup analyses revealed post-intervention effects of lifestyle PA and SB interventions on steps, fatigue, disease activity and functional ability, however, these were not sustained at follow-up. No post-intervention effects were observed for quality of life and pain, although follow-up effects on these outcomes were seen. Both post-intervention and follow-up effects were demonstrated for light/leisure PA only. The varied results regarding intervention efficacy at different assessment timepoints may be due to follow-up periods being particularly heterogeneous between studies (ranging from 6 months to 4 years). A more consistent approach between interventions would give greater insight into the long-term effectiveness of these interventions. A considerable number of interventions (*n* = 9) included in this review did not conduct follow-up assessments. Therefore, it is not surprising that little is known regarding the effectiveness of interventions to promote long-term adherence to PA and SB. By necessitating that follow-up assessments are done, this ensures interventions are targeting sustained clinical benefits [17, 57]. In addition, interventions which demonstrate beneficial effects at long-term follow-up (i.e., 4 years post-intervention) reflect a more sustained lifestyle change, whereby adoption evolves into maintenance [52]. Previous reviews and qualitative findings have reported that a main challenge of an intervention program is to assess and ensure beneficial effects post-intervention [19, 57, 65]. Therefore, future interventions should conduct regular follow-up assessments over long periods, to assess their long-term clinical efficacy.

Compared to multi-component interventions, interventions that focused primarily on promoting PA or reducing SB, were more successful in terms of number and relative size of observed statistically significant improvements in behaviours (increased PA and/or reduced SB) and outcomes. In turn, where these focused interventions demonstrated increased PA and/or reduced SB, greater improvements were also observed in disease activity, functional ability, pain and fatigue in particular. A common feature of interventions primarily targeting activity behaviours was that they frequently reported information pertaining to the “dose” of the intervention. For example, these interventions reported details regarding the PA type, intensity, frequency and duration delivered in the intervention [21, 42, 53], whereas multi-component interventions typically provided a vague behavioural goal (e.g., information on benefits of PA and teaching of a home-based exercises [25]). This reporting may have helped participant adherence, improved the accuracy and clarity of findings, and increased understanding the effects of specific PA dosages on specific outcomes [18].

Such PA/SB focused interventions were also often more personalised and tailored to individuals’ abilities and had good adherence [21, 42, 53]. Moreover, these interventions may be deemed more feasible by people with RA, who have additional disease-related barriers to PA [17], leading to more successful implementation and potential effects. Our present results support findings of a previous meta-analysis in healthy adults [66]. By contrast, this review found that multi-component interventions (e.g., including counselling, education, nutrition advice and/or self-management), targeting multiple health behaviours (i.e., not primarily focused on promoting PA or reducing SB) with less information about PA “dosage”, appeared to be less effective, with fewer improvements in health, increases in PA and/or reductions in SB. This finding may suggest that interventions that include a primary focus on lifestyle PA and/or SB, appear more effective than multi-component interventions, and we suggest future multi-component interventions provide more detailed PA/SB guidance or prescription for RA participants if their aim is to improve activity behaviours. However, whilst this review provides the first novel insight into the relative effectiveness of single (i.e., targeting PA/SB) vs. multi-component interventions for promoting PA and/or reducing SB, these comparisons are beyond the scope of this review, and will be an important focus of future research.

Successful interventions also included regular support, most commonly in the form of text messages [21], regular phone calls at a frequency of every 1 to 2 weeks [8, 42, 43], or individualised based on goals [21, 48]. However, frequency and type of support varied across studies. Future research could explore what mode and frequency of support is likely to be optimal for this patient group, and behavioural support components should be further explored as a potential intervention aid in future trials in people with RA. Successful interventions were also more likely to be delivered in accessible settings, rather than a specified facility (e.g., public training centre, gym), which has previously shown to be an obstacle for intervention adherence [8, 39, 42]. Those resource intensive interventions included in this review were generally more multi-component in nature, and required travel to other settings for the other components of their interventions [8]. Perhaps the complex nature, and focus on multiple health behaviours of some multi-component interventions, diluted down the key message of lifestyle PA and SB interventions, to simply move more.

Interventions where the primary focus was on promoting PA or reducing SB generally employed devices (e.g., accelerometers), to assess specific individual dimensions or domains of PA and/or SB (i.e., frequency, intensity, time or type of PA, or total or patterns of sedentary time (e.g., bouts, breaks)). The apparent effectiveness of interventions using device-based measures, relative to those employing self-report, may have been partially due to the increased validity and reliability of device-based measures compared to questionnaires. Indeed, self-report methods are subject to recall bias, and this may explain why no effects were observed for total PA outcome which was most frequently assessed using questionnaires (e.g., International Physical Activity Questionnaire). In addition, device-based and self-report measures of PA and SB are not conceptually equivalent, producing different outputs, and offer different approaches to measure PA and SB [67]. Therefore, future research should examine interventions which use device-based vs self-report measures of PA or SB separately in meta-analyses, when a greater number of high-quality studies have been conducted. Due to lack of evidence currently available from the studies included in this review, we could not confidently group studies this way without introducing a degree of bias, and so we were unable to do this subgroup analysis.

### Strengths and limitations

Strengths include the use of transparent methods including pre-registration, clear inclusion criteria and a robust search strategy; and therefore, results and conclusions are likely to be valid and can be replicated in future reviews. The subgroup analysis allowed for the exploration of moderating variables, to give more investigative interpretation of results, while GRADE analysis allowed for assessing the quality of evidence. Lastly, our choice of core OMERACT outcomes to describe RA-related health helped to identify gaps in current research, which should be addressed in future interventions.

In meta-analyses, functional ability and depression outcomes could not be successfully transformed, so were split into normal and non-normal outcomes which gave different results. Therefore, findings regarding these outcomes should be interpreted with caution. Moreover, no subgroup analyses were undertaken for mode of intervention delivery (e.g., individual, group, internet, app-based), dimension of lifestyle PA/SB targeted, nature of the comparison group (e.g., placebo, no intervention, advice only), and whether interventions had a theoretical basis. This was due to heterogeneity between studies; meaning we were unable to confidently group studies into these categories. This heterogeneity was also apparent between overall intervention content and structure. This, however, was taken into account in GRADE analysis and therefore, our conclusions are drawn in perspective of the final quality of evidence and thus, consider heterogeneity. Our study is the first to shed light on the value of the interventions targeting lifestyle PA and SB for improving RA health outcomes. This review and meta-analysis is therefore a step in the right direction to guide more research in this area, so we can start to determine specifically what components of interventions are most effective, and for whom they are effective given the varying nature of RA disease activity between individuals.

In addition, study participants were heterogenous, and most had low disease activity and few severe disabilities. There was also little information provided in papers regarding treatment pathways of participants (e.g., Disease Modifying Anti-Rheumatic Drugs vs escalation to biologic therapies). Therefore, we are unable to draw conclusions based on our findings for these RA subpopulations. We recommend that future lifestyle PA and/or SB interventions should specifically target these subpopulations with greater levels of disability, higher disease activity, and considering their treatment pathways.

### Implications

Future interventions should be clearer and more specific in describing subgroups for meta-analyses to be able to assess their efficacy at improving core OMERACT patient- and clinician-important outcomes in people with RA. Therefore, future studies should publish trial registrations or protocols, provide information about participant and personnel blinding, and use validated measures to assess outcomes to ensure transparent reporting of results. Moreover, small-scale feasibility interventions were included in this review which were not adequately powered to detect statistically significant changes in outcomes. Nevertheless, conducting feasibility studies shows good research practice, and future large-scale interventions using identical study designs and methods are welcomed to confirm and strengthen their findings.

The choice of outcomes was varied and inconsistent between studies, showing little consideration of OMERACT guidelines [28]. There was also little consistency between outcome measurement methods, as demonstrated by the high I^2^ statistic results for many meta-analyses which negatively influenced and downgraded GRADE analysis results. Consequently, GRADE analysis results displayed “very low” and “low” study quality for functional ability (normal), pain, anxiety, depression (normal and non-normal), quality of life and sedentary time outcomes (Table 2). Therefore, results for these outcomes should be interpreted with caution.

Accordingly, researchers need to provide a consensus on the optimal methods and outcomes to reliably assess the efficacy of lifestyle PA and SB interventions in the RA population. As studies consistently displayed moderate to high risk of bias (Figs. 3a and 4), as well as heterogenous quality results (GRADE analysis, Table 2), future investigations should seek to provide more detailed explanations of study design and methods to enable researchers to replicate and strengthen these findings (i.e., by accounting for and reducing between-study heterogeneity). Concerning, measurement of risk of bias, the RoB2 tool used in this study is the most used and recommended tool for use by the Cochrane Handbook [36]. However, studies have reported poor to moderate agreement between RoB2 and other quality appraisal scales (e.g., the PEDRO scale) [68, 69], suggesting the choice of tool may impact the validity of our results in this regard. However, many interventions included in this study did not report key risk of bias criteria, resulting in moderate to high risk of bias being observed. As such, it is unlikely that using another tool would have altered our conclusion. Still, experts recommend a consistent approach should be adopted with risk of bias tools not used interchangeably within systematic reviews, and as such, high-quality, validated risk of bias tools (such as the RoB2), should be used to ensure consistency in quality recommendations in future systematic reviews [69].

### Conclusions

We detected that lifestyle PA and SB interventions increased certain dimensions of PA, as well as improved disease activity and other core OMERACT patient- and clinician-important outcomes in people with RA. PA and SB interventions differed in effectiveness at targeting different outcomes, due to differences in content, structure and focus of the intervention, demonstrated by varied results for different outcomes in GRADE analysis findings. In addition, due to differing follow-up assessment periods, intervention benefits on outcomes at post-intervention and follow-up were inconsistent. Future research in this area should seek to standardise PA, SB and health outcome measures and measurement tools across studies, and employ regular/consistent follow-up periods to allow clinical benefit of interventions to be assessed. More studies are also required to explore the value of interventions targeting SB for improving health in RA.

### Supplementary Information

**Additional file 1: Supplementary Materials: Supplementary Table 1.** PICO question and criteria. **Supplementary Table 2.** Search Strategies for 8 databases. **Supplementary Figures 1-13.** Forest plots for secondary outcomes- Physical Activity vs Sedentary Behaviour interventions. **Supplementary Figures 14-26.** Forest plots for secondary outcomes- Post-intervention vs follow-up. **Supplementary Figures 27-35.** Funnel plots for meta-analyses with 10+ entries.

## Data Availability

All data generated or analysed during this study are included in this published article [and its supplementary information files].

## Abbreviations

RA: Rheumatoid Arthritis
PA: Physical Activity
SB: Sedentary Behaviour
MVPA: Moderate-to-vigorous PA
OMERACT: Outcome Measures in Rheumatoid Arthritis Clinical Trials
PROSPERO: Prospective Register of Systematic Review
PRISMA: Preferred Reporting Items for Systematic Reviews and Meta-Analyses
CINAHL: Cumulative Index to Nursing & Allied Health Literature
EMBASE: Excerpta Medica database
PEDro: Physiotherapy Evidence Database
RCT: Randomised controlled trial
RoB2: Cochrane Risk of Bias 2 tool
NIH: National Institute of health
GRADE: Grading of Recommendations Assessment Development and Evaluation
MD: Mean difference
SMD: Standardised mean difference
CI: Confidence interval
DAS28: Disease activity score 28
RADAI: Rheumatoid Arthritis Disease Activity Index
CDAI: Clinical Disease Activity Index
HADS: Hospital Anxiety and Depression Scale
HAQ: Stanford Health Assessment Questionnaire
MCID: Minimal clinically important difference
