# Supplementary material for: Effects of lifestyle physical activity and sedentary behaviour interventions on disease activity and patient- and clinician- important health outcomes in rheumatoid arthritis: a systematic review with meta-analysis
Source: BMC Rheumatol. 2023 Sep 6;7:27. doi: 10.1186/s41927-023-00352-9 (PMC10481589; doi:10.1186/s41927-023-00352-9)

**Supplementary Materials**

# Title: EFFECTS OF LIFESTYLE PHYSICAL ACTIVITY AND SEDENTARY BEHAVIOUR INTERVENTIONS ON DISEASE ACTIVITY AND PATIENT- AND CLINICIAN- IMPORTANT HEALTH OUTCOMES IN RHEUMATOID ARTHRITIS: A SYSTEMATIC REVIEW WITH META-ANALYSIS

**Journal**: Sports Medicine

**Authors**: Sophia M. Brady^1,2,3^, Jet J.C.S. Veldhuijzen van Zanten^1,2,3^, Petros C. Dinas,^4^ Tom E. Nightingale^1,5^, George S. Metsios^6^, Saleh M.A. Elmsmari^1^, Joan L. Duda^1,7^, George D. Kitas^1,2^, Sally A.M. Fenton^1,2,3^

**Corresponding Author**

Sally A.M. Fenton

School of Sport, Exercise and Rehabilitation Sciences

University of Birmingham

Birmingham

B15 2TT

United Kingdom

[s.a.m.fenton@bham.ac.uk](mailto:s.a.m.fenton@bham.ac.uk)

**Co-Authors:**

Sophia M. Brady (SB)

Jet Veldhuijzen van Zanten (JVvZ)

Petros Dinas (PCD)

Tom E. Nightingale (TN)

George Metsios (GS)

Saleh M.A. Elmsmari (SE)

Joan L. Duda (JD)

George Kitas (GK)

Sally A.M. Fenton (SF)

^1^ School of Sport, Exercise and Rehabilitation Sciences, University of Birmingham, Birmingham, United Kingdom

^2^ Rheumatology Department, Dudley Group NHS Foundation Trust, Dudley, United Kingdom

^3^ Medical Research Council- Versus Arthritis Centre for Musculoskeletal Ageing, University of Birmingham, Birmingham, United Kingdom

^4^ FAME Laboratory, Department of Physical Education and Sport Science, University of Thessaly, Thessaly, Greece

^5^ Centre for Trauma Science Research, University of Birmingham, Birmingham, United Kingdom

^6^ Department of Nutrition and Dietetics, School of Physical Education, Sport Science and Dietetics, University of Thessaly, Thessaly, Greece

^7^ Institute of Mental Health, University of Birmingham, Birmingham, United Kingdom

***Supplementary Table 1: PICO question and criteria***

| ***PICO*** | ***Inclusion and Exclusion Criteria*** |
| --- | --- |
| **Population** | **Inclusion criteria:**   - Any adult patient population with a consultant- or self-diagnosis of Rheumatoid Arthritis (RA) irrespective gender, ethnicity or socioeconomic background with early or long-standing RA, under any treatment (non-steroidal anti-inflammatory drugs, steroids, disease-modifying anti-rheumatic drugs including biologics), will be eligible for this study.   **Exclusion criteria:**   - Studies involving a mixed sample of patients with inflammatory joint conditions will be excluded when the effects of the lifestyle physical activity (PA) or sedentary behaviour (SB) interventions cannot be isolated specifically for RA patients (i.e., when the publication does not separate the effects of the intervention into the different groups of patients with inflammatory joint conditions). |
| **Intervention** | **Inclusion criteria:**   - Physical activity (PA) is defined as any bodily movement which results in skeletal muscle contraction and increases in energy expenditure. We are specifically looking at interventions which promote lifestyle physical activity, not organized, structured exercise. Lifestyle PA is the accumulation of PA through planned or unplanned leisure, household, occupation or transport, as part of everyday life. We will include interventions targeting and measuring lifestyle PA accumulated across all life domains. - Sedentary Behaviour (SB) is defined as “*any waking behaviour requiring energy expenditure ≤1.5 metabolic equivalents and a sitting or reclining posture*” according to the Sedentary Behaviour Research Network. It comprises several different behaviours (e.g., TV viewing, computer use, driving), and can be accumulated across different life domains (e.g., during leisure time, home, community, occupation and transport). We will include interventions targeting any SBs across all life domains. - Interventions which directly target promoting lifestyle PA and reducing SB as part of the intervention. - The intervention mode has to target lifestyle PA and SB, but that doesn’t have to be the primary outcome. However, lifestyle PA and/or SB have to be reported as an outcome (can be a secondary outcome). - Multi-component interventions (i.e., which focused on other behaviours alongside PA, such as diet), were included if they; 1) included a component focused on lifestyle PA and/or SB, and 2) measured PA and/or SB as an outcome. - Any lifestyle PA or SB intervention delivered in any setting (e.g., across all domains –at home, community, occupation, leisure and transport). - Any lifestyle PA interventions targeting overall lifestyle PA, or specific modes of activity through which lifestyle PA can be accumulated; such as walking, gardening, active transport, stair climbing. - Any SB interventions involving reducing overall SB or breaking up SB; such as reducing TV viewing, using a standing desk at work. - Any mode of delivery (e.g. groups vs. individuals, delivered online, in clinic) - Interventions published in English, before August 2022.   **Exclusion criteria:**   - Multi-component interventions will be excluded, if they do not include a lifestyle PA and/or SB component in their intervention and also measure PA and/or SB as an outcome. - Overall arthritis self-management programmes, which do not directly target lifestyle PA or SB as part of the intervention. - Exercise is defined, by the World Health Organisation, as planned, structured, repetitive and intentional movement with the objective to improve and/or maintain fitness. We will exclude interventions that involve only prescribed, purposeful, structured exercise interventions, as these do not fall under the category of lifestyle PA. However, interventions which include structured exercise as part of an intervention promoting overall lifestyle PA, will be included. - Home based exercise programs and studies which are based on the effects of physiotherapy (range of motion exercises, therapeutic exercise regimens) and trials restricted to addressing physical impairments. |
| **Comparison** | We will compare patients that undertake an intervention with patients in all the groups described below:   1. no lifestyle PA or SB (no intervention) 2. usual care 3. placebo intervention (i.e., interventions which do not affect the main outcome of lifestyle PA or SB, such as nutritional intervention, medication or information on RA) 4. PA or SB advice at baseline but no intervention (e.g., baseline information from healthcare professional, leaflet, video)   A *priori* will be given to randomized controlled trials, but controlled trials and quasi-randomized controlled trials will also be considered for inclusion in this review. |
| **Outcomes** | For the identification of our primary and secondary outcomes, we have considered in our decisions: a) harmonising our outcomes with outcomes of relevant reviews in the Cochrane Library (as per Cochrane handbook), and b) importance of outcomes to clinicians, patients and policy makers, based on the core sets of outcomes outlined by both OMERACT and COMET (which both inform the content of the Cochrane Library). Therefore, we aim to assess the effects of lifestyle PA and SB on the following primary and secondary outcomes:  What are the major outcomes?   1. **Patient important outcomes** - quality of life, pain (and stiffness), mobility, fatigue, independence, sleep, anxiety, depression 2. **Clinician important outcomes** - disease activity (DAS28), number of tender/swollen joints, physical function/functional ability, disease severity (HAQ), structural joint damage, inflammatory markers (CRP, ESR), comorbidity risk (e.g., for CVD), changes in medication (dose and type), adverse events. 3. Lifestyle PA (change from baseline) 4. Lifestyle SB (change from baseline)   What subgroup analyses do you intend to undertake?  We intend to investigate the effects of the following different types of interventions:   1. Mode of delivery - e.g., setting (individual, group, internet, app-based) 2. Theory based vs. non-theory based interventions (e.g., self-efficacy, attitude, autonomy) 3. Type of lifestyle PA promoted/SB targeted in the intervention 4. Domain of lifestyle PA/SB targeted (e.g., overall vs. leisure time) 5. Outcome assessment (self-reported vs. device-assessed PA and SB) 6. Comparison group (placebo, no intervention, advice only). 7. Consultant vs. self-diagnosed RA participants used in study |

***Supplementary Table 2: Search Strategies for 8 databases***

| ***Database*** | ***Search Strategy*** |
| --- | --- |
| ***Cochrane Library*** | ("rheumatoid arthritis") AND ("physical activity" OR Physical Fitness OR "physical function" OR "Activities of Daily Living" OR "lifestyle physical activity" OR "life-style physical activity" OR Motor Activity OR "lifestyle behavior" OR "lifestyle behaviour" OR sedentary OR “Sedentary Behavior” OR “sedentary lifestyle” OR "sedentary behaviour" OR sitting OR "sitting time" OR "sitting behavior" OR screen time) AND (randomized controlled trial OR controlled clinical trial OR Random Allocation OR "randomised controlled trial" OR randomized OR single-blind method OR Double-Blind Method OR trial OR groups OR intervention OR placebo) AND (promot* OR educat* OR uptake OR start OR increase OR program*) |
| ***CINAHL Plus*** | ("rheumatoid arthritis") AND ("physical activity" OR “Physical Fitness” OR "physical function" OR "Activities of Daily Living" OR "lifestyle physical activity" OR "life-style physical activity" OR “Motor Activity” OR "lifestyle behavior" OR "lifestyle behaviour" OR “sedentary” OR “Sedentary Behavior” OR “sedentary lifestyle” OR "sedentary behaviour" OR “sitting” OR "sitting time" OR "sitting behavior" OR “screen time”) AND (“randomized controlled trial” OR “controlled clinical trial” OR “Random Allocation” OR "randomised controlled trial" OR “randomized” OR “randomised” OR “single-blind method” OR “Double-Blind Method” OR “trial” OR “groups” OR “intervention” OR “placebo”) AND (“promot*” OR “educat*” OR “uptake” OR “start” OR “increase” OR “program*”) |
| ***Scopus*** | ABS ( "rheumatoid arthritis" ) AND ( "physical activity" OR physical AND fitness OR "physical function" OR "activities of daily living" OR "lifestyle physical activity" OR "life-style physical activity" OR motor AND activity OR "lifestyle behavior" OR "lifestyle behaviour" OR sedentary OR sedentary AND behavior OR sedentary AND lifestyle OR "sedentary behaviour" OR sitting OR "sitting time" OR "sitting behavior" OR screen AND time ) AND ( randomized AND controlled AND trial OR controlled AND clinical AND trial OR random AND allocation OR "randomised controlled trial" OR randomized OR single-blind AND method OR double-blind AND method OR trial OR groups OR intervention OR placebo ) AND ( promot* OR educat* OR uptake OR start OR increase OR program* ) AND ( LIMIT-TO ( DOCTYPE , "ar" ) ) |
| ***PEDro*** | “rheumatoid arthritis” filter by clinical trials AND health promotion/fitness training/behaviour modification/education |
| ***PsychINFO, Medline, EMBASE*** | "rheumatoid arthritis"  “physical activity" OR Physical Fitness OR "physical function" OR "Activities of Daily Living" OR "lifestyle physical activity" OR "life-style physical activity" OR Motor Activity OR "lifestyle behavior" OR "lifestyle behaviour" OR sedentary OR “Sedentary Behavior” OR “sedentary lifestyle” OR "sedentary behaviour" OR sitting OR "sitting time" OR "sitting behavior" OR screen time  randomized controlled trial OR controlled clinical trial OR Random Allocation OR "randomised controlled trial" OR randomized OR single-blind method OR Double-Blind Method OR trial OR groups OR intervention OR placebo  promot* OR educat* OR uptake OR start OR increase OR program* |
| ***Web of Science*** | TS= ("rheumatoid arthritis") AND TS=(“physical activity" OR “Physical Fitness” OR "physical function" OR "Activities of Daily Living" OR "lifestyle physical activity" OR "life-style physical activity" OR “Motor Activity” OR "lifestyle behavior" OR "lifestyle behaviour" OR “sedentary” OR “Sedentary Behavior” OR “sedentary lifestyle” OR "sedentary behaviour" OR “sitting” OR "sitting time" OR "sitting behavior" OR “screen time”) AND TS=(“randomized controlled trial” OR “controlled clinical trial” OR “Random Allocation” OR "randomised controlled trial" OR “randomized” OR “single-blind method” OR “Double-Blind Method” OR “trial” OR “groups” OR “intervention” OR “placebo”) AND TS=(“promot*” OR “educat*” OR “uptake” OR “start” OR “increase” OR “program*”) |

Note: CINAHL= Cumulative Index to Nursing & Allied Health Literature, EMBASE= Excerpta Medica database, PEDro= Physiotherapy Evidence Database

***Supplementary Figures 1-13: Forest plots for secondary outcomes- Physical Activity vs Sedentary Behaviour interventions***


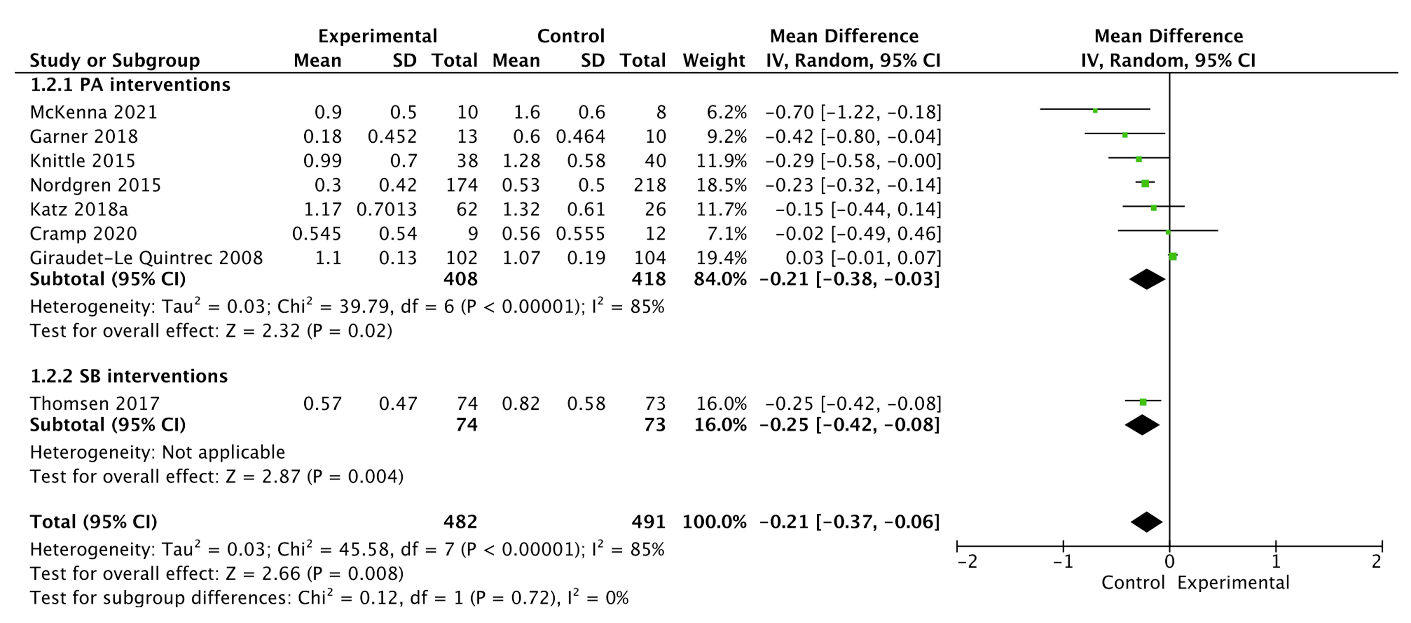


*Supplementary Figure 1: The effects of interventions on functional ability (normally distributed). SD= standard deviation, 95% CI= 95% confidence interval.*

2007

*Supplementary Figure 2: The effects of interventions on functional ability (non-normally distributed). SD= standard deviation, 95% CI= 95% confidence interval.*


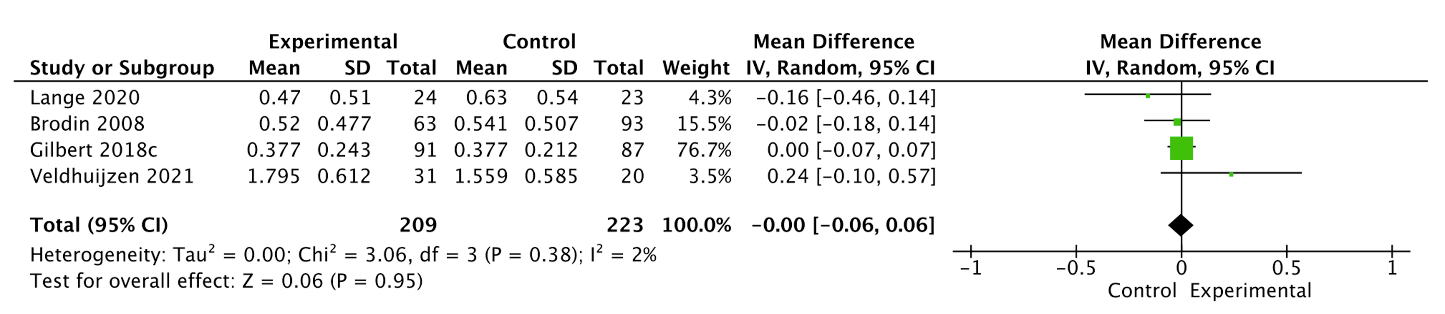


*Supplementary Figure 3: The effects of interventions on pain. SD= standard deviation, 95% CI= 95% confidence interval.*


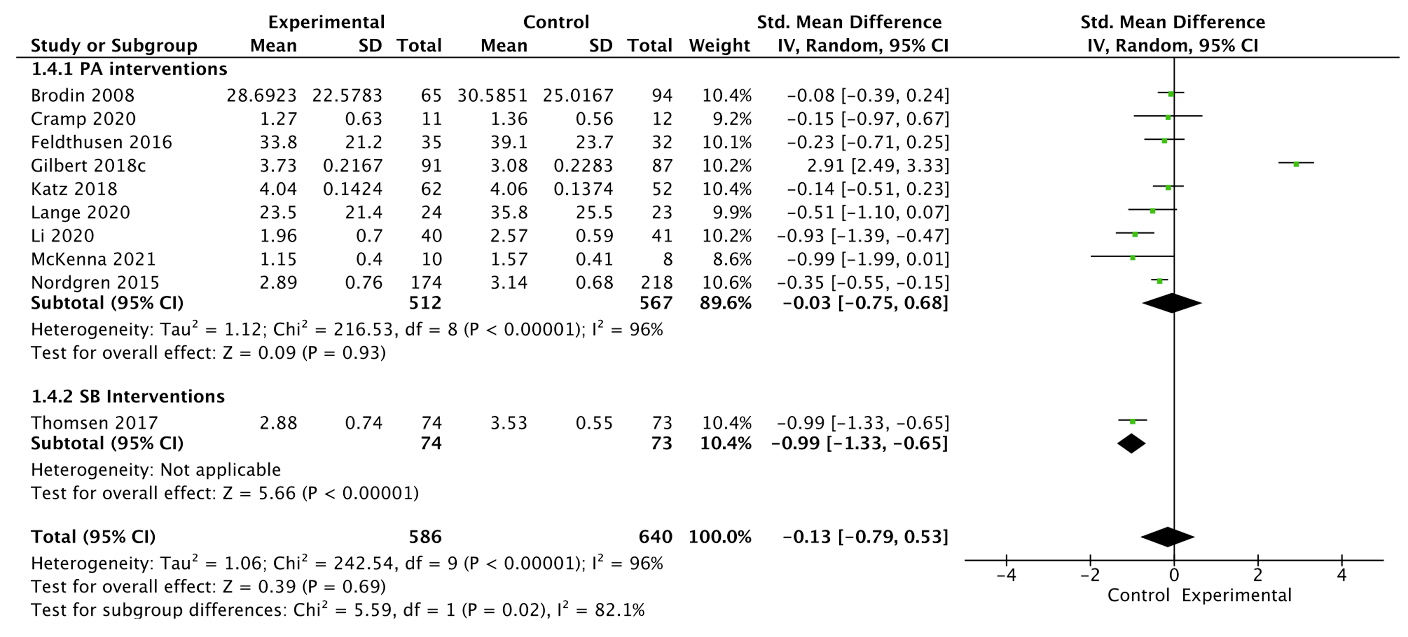


*Supplementary Figure 4: The effects of interventions on fatigue. SD= standard deviation, 95% CI= 95% confidence interval.*


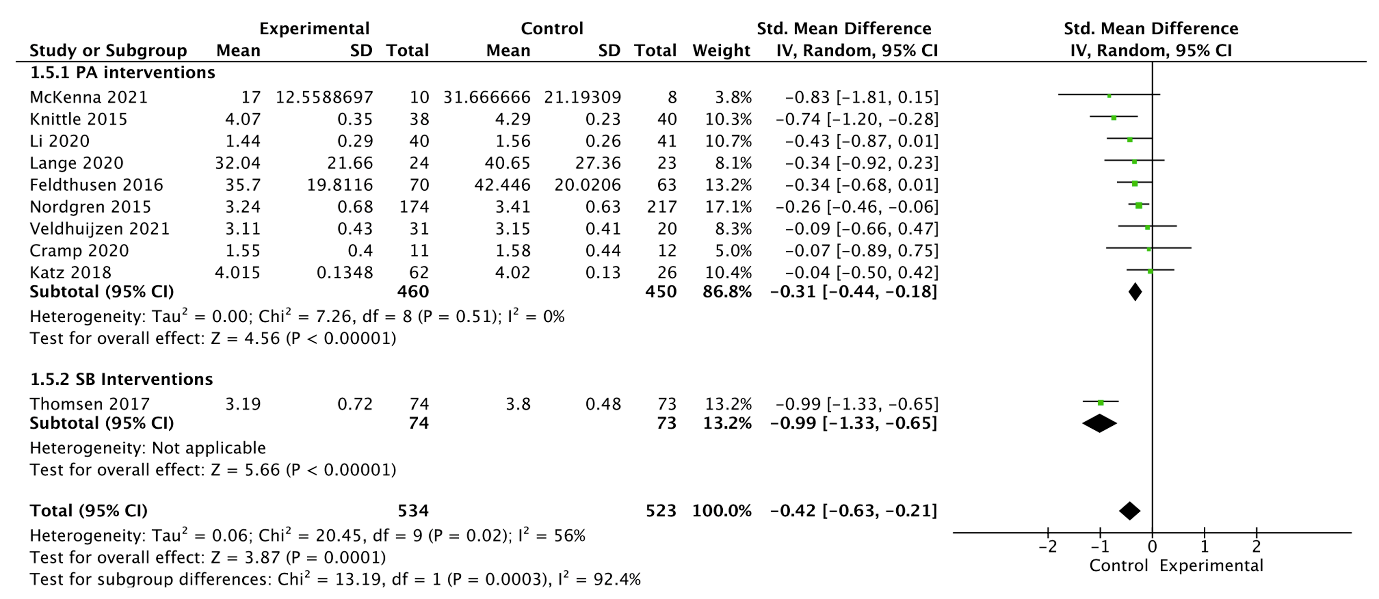


*Supplementary Figure 6: The effects of interventions on depression (non-normally distributed). SD= standard deviation, 95% CI= 95% confidence interval.*


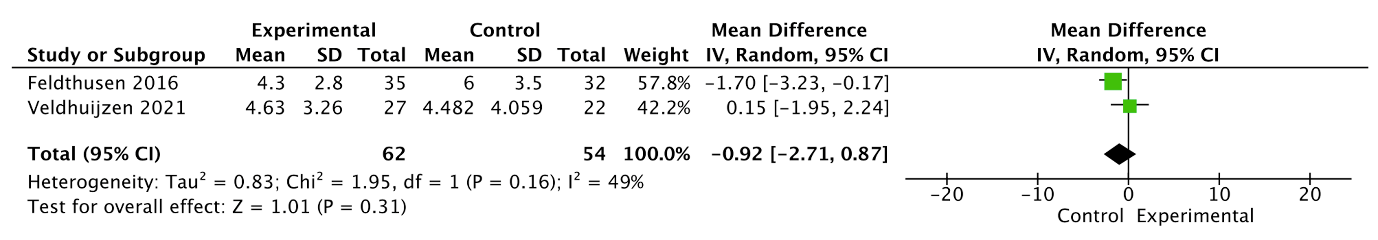


*Supplementary Figure 7: The effects of interventions on depression (normally distributed). SD= standard deviation, 95% CI= 95% confidence interval.*


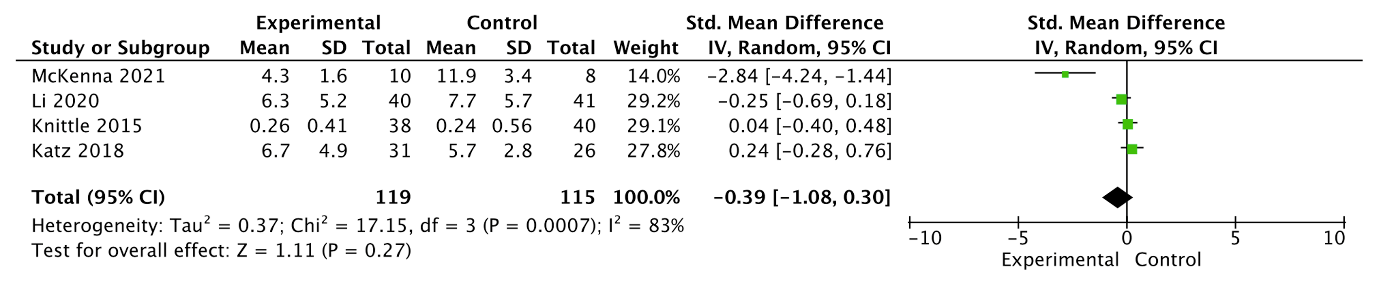


*Supplementary Figure 5: The effects of interventions on anxiety. SD= standard deviation, 95% CI= 95% confidence interval.*


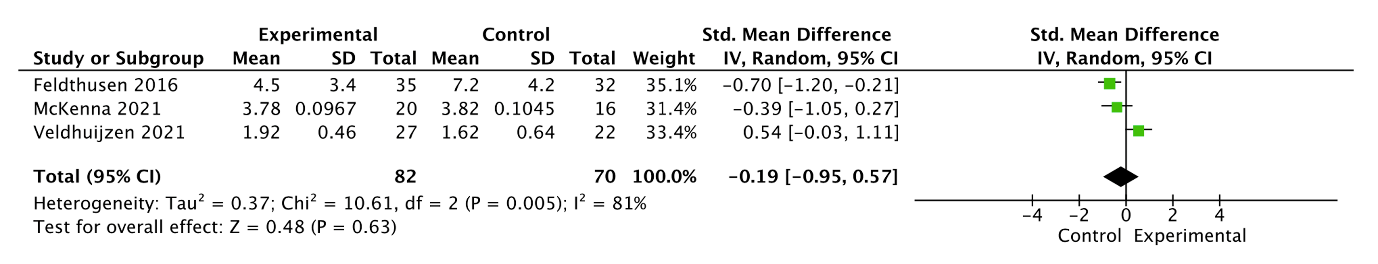


*Supplementary Figure 9: The effects of interventions on sedentary time. SD= standard deviation, 95% CI= 95% confidence interval.*


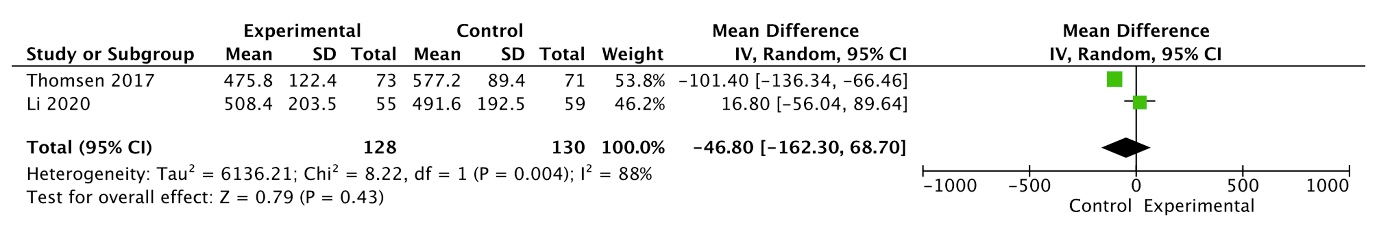


*Supplementary Figure 8: The effects of interventions on quality of life. SD= standard deviation, 95% CI= 95% confidence interval.*


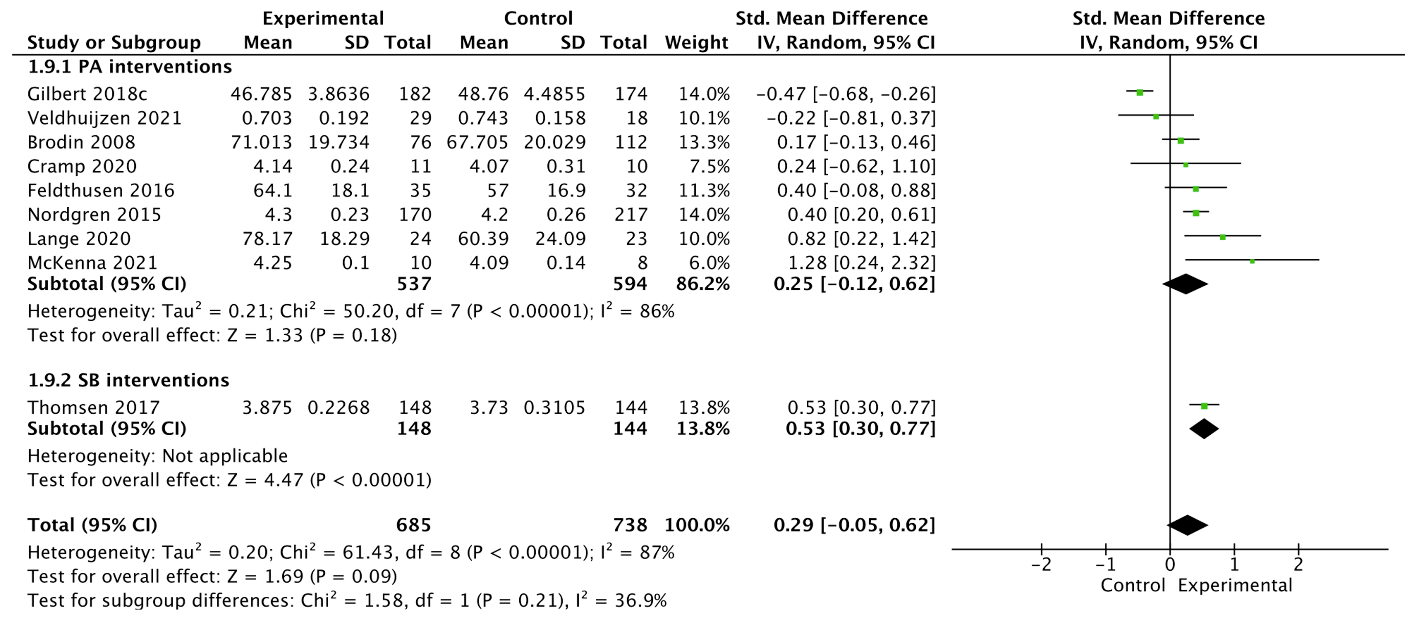


*Supplementary Figure 10: The effects of interventions on daily steps. SD= standard deviation, 95% CI= 95% confidence interval.*


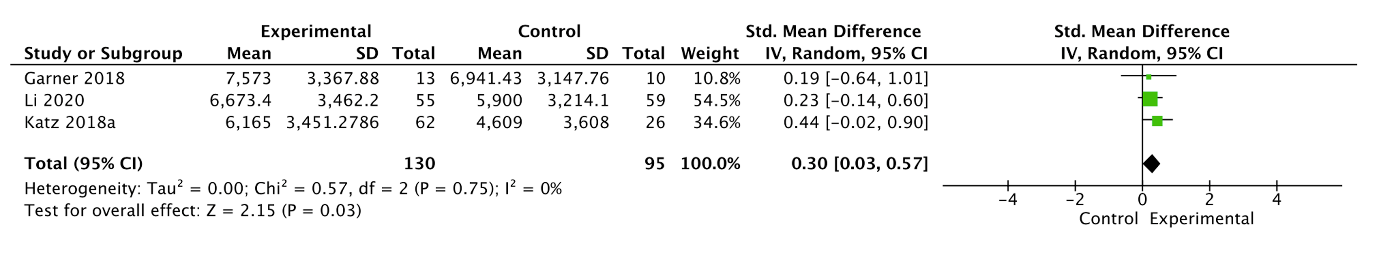


*Supplementary Figure 11: The effects of interventions on moderate to vigorous physical activity. SD= standard deviation, 95% CI= 95% confidence interval.*


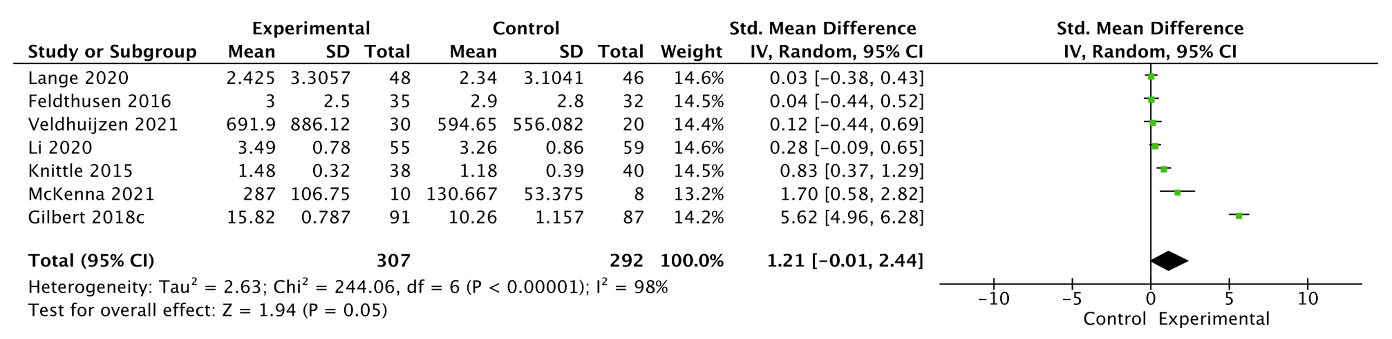


*Supplementary Figure 12: The effects of interventions on total physical activity. SD= standard deviation, 95% CI= 95% confidence interval.*


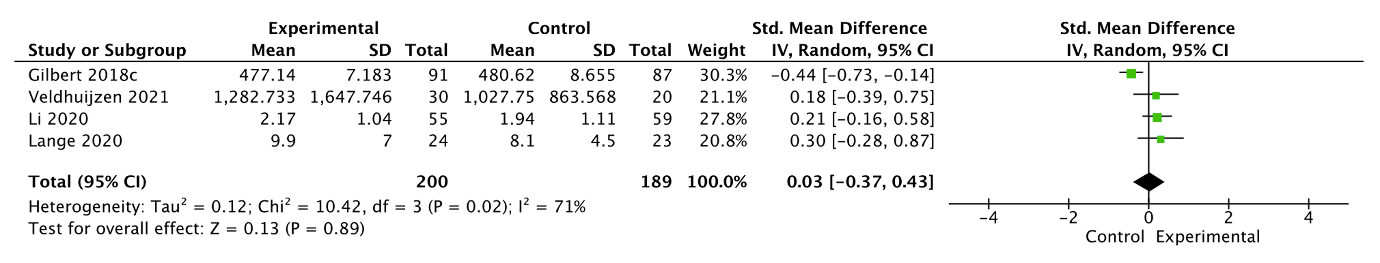


*Supplementary Figure 13: The effects of interventions on leisure/light* *intensity physical activity. SD= standard deviation, 95% CI= 95% confidence interval.*


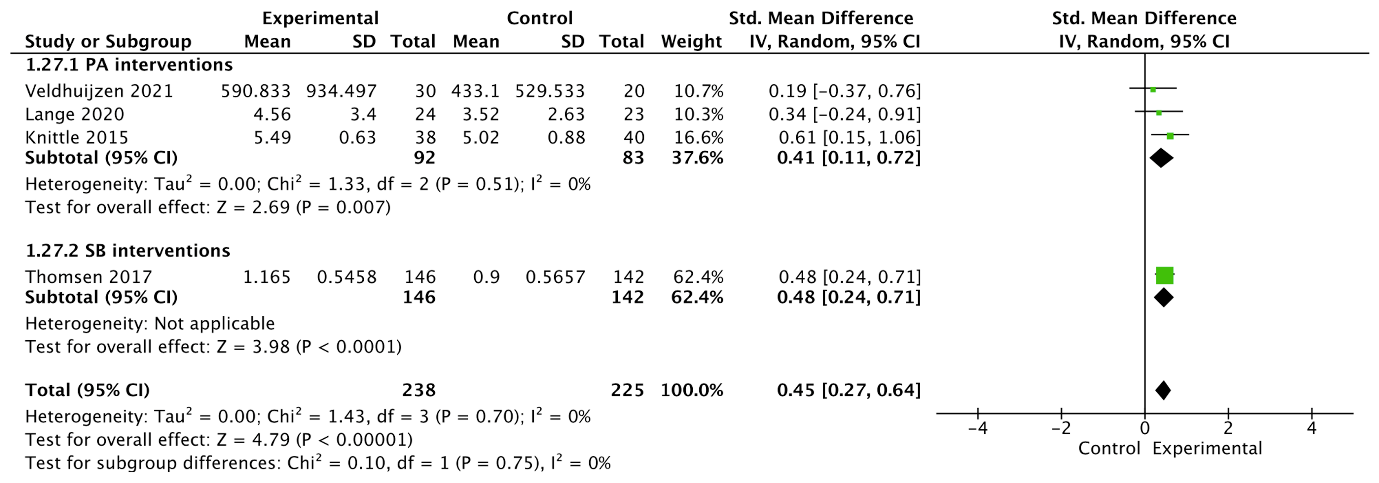


***Supplementary Figures 14-26: Forest plots for secondary outcomes- Post-intervention vs follow-up***

*Supplementary Figure 15: The effects of interventions at post-intervention and follow-up for functional ability (non-normal). SD= standard deviation, 95% CI= 95% confidence interval.*


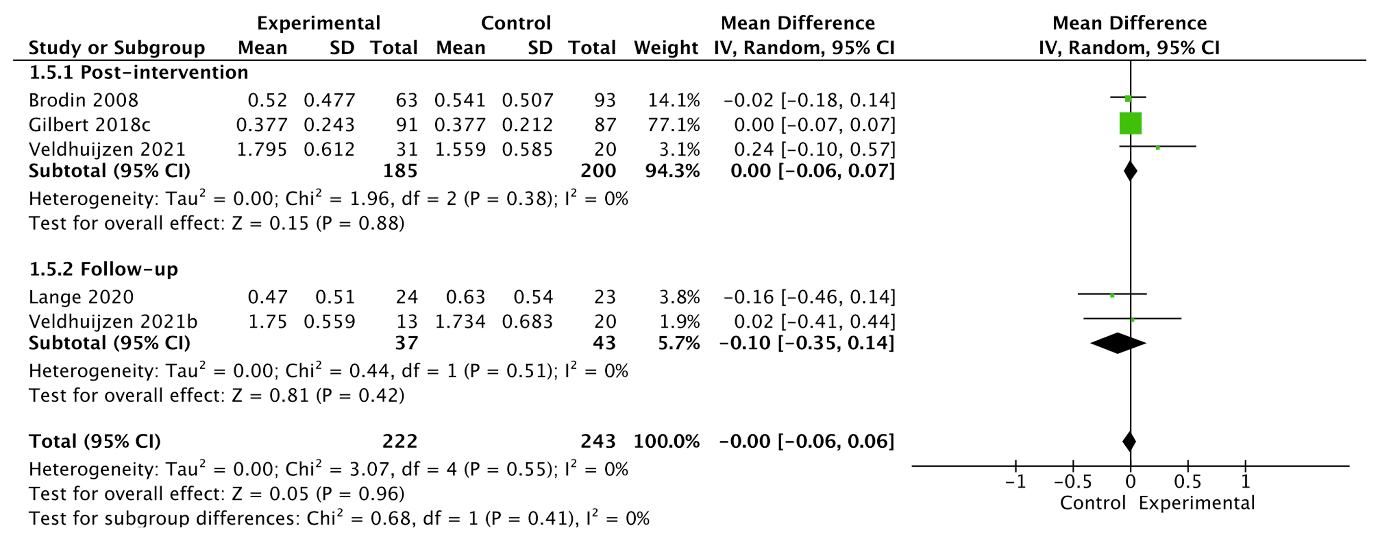


*Supplementary Figure 14: The effects of interventions at post-intervention and follow-up for functional ability (normally distributed). SD= standard deviation, 95% CI= 95% confidence interval.*


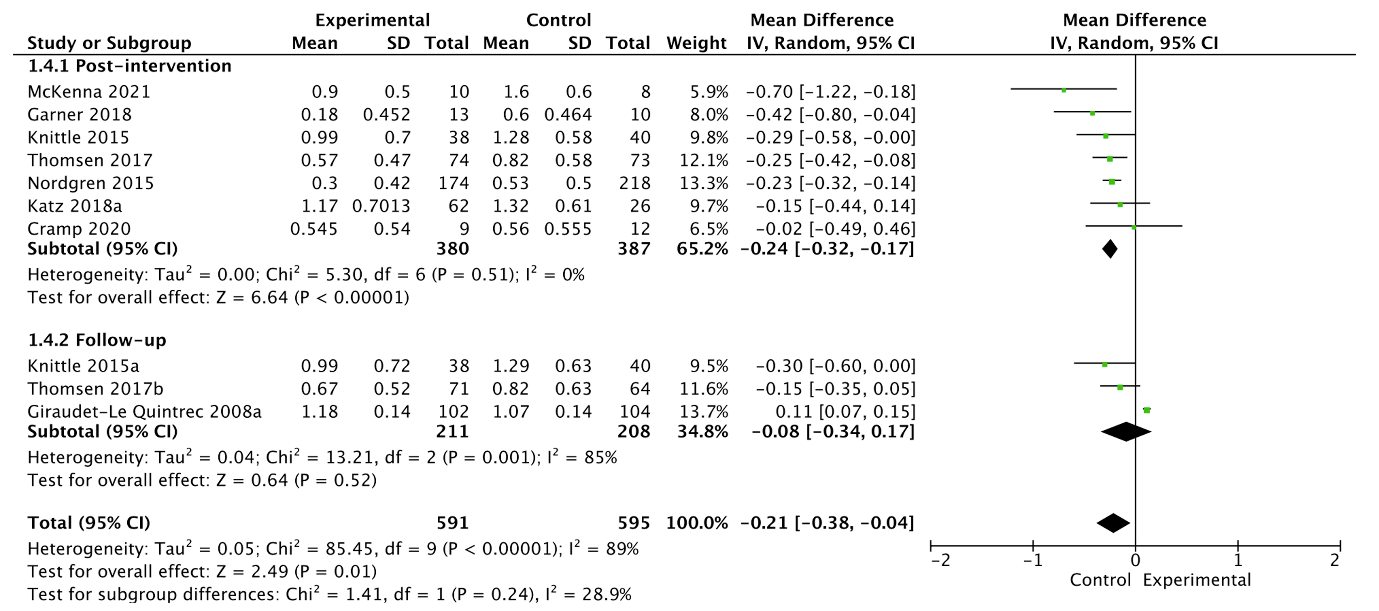


2007

*Supplementary Figure 16: The effects of interventions at post-intervention and follow-up pain. SD= standard deviation, 95% CI= 95% confidence interval.*


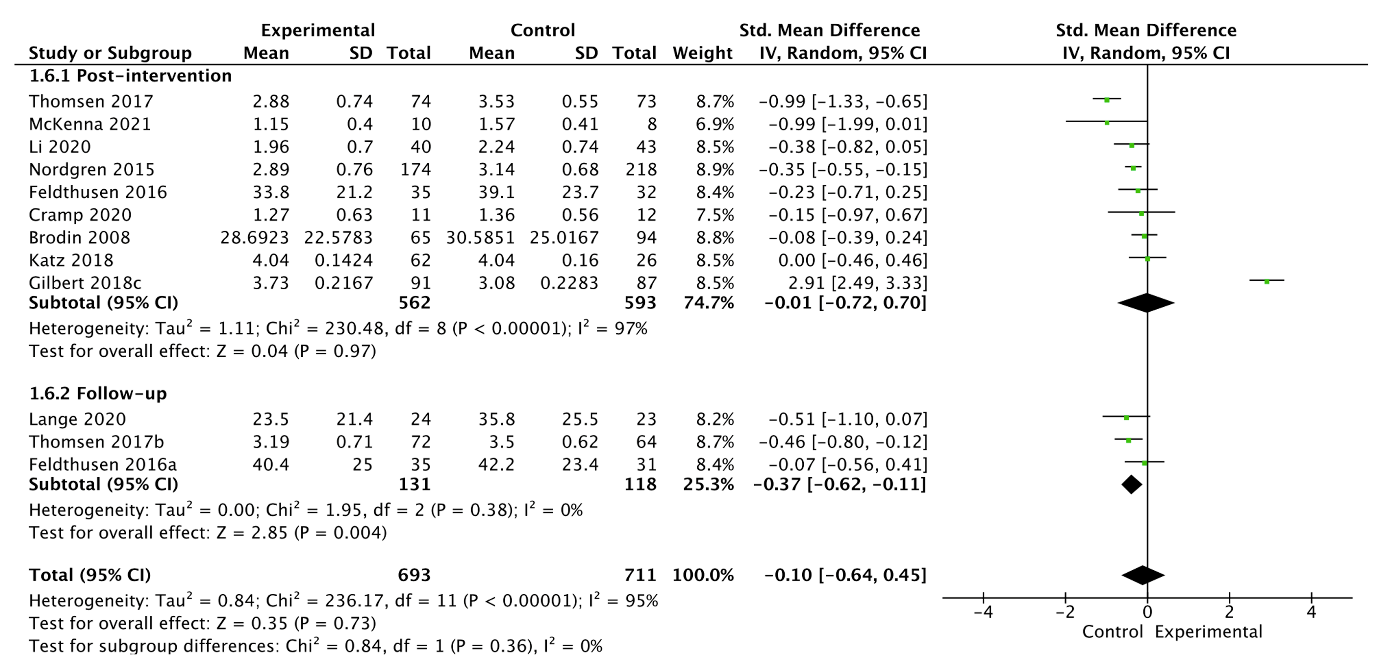


*Supplementary Figure 17: The effects of interventions at post-intervention and follow-up for fatigue. SD= standard deviation, 95% CI= 95% confidence interval.*


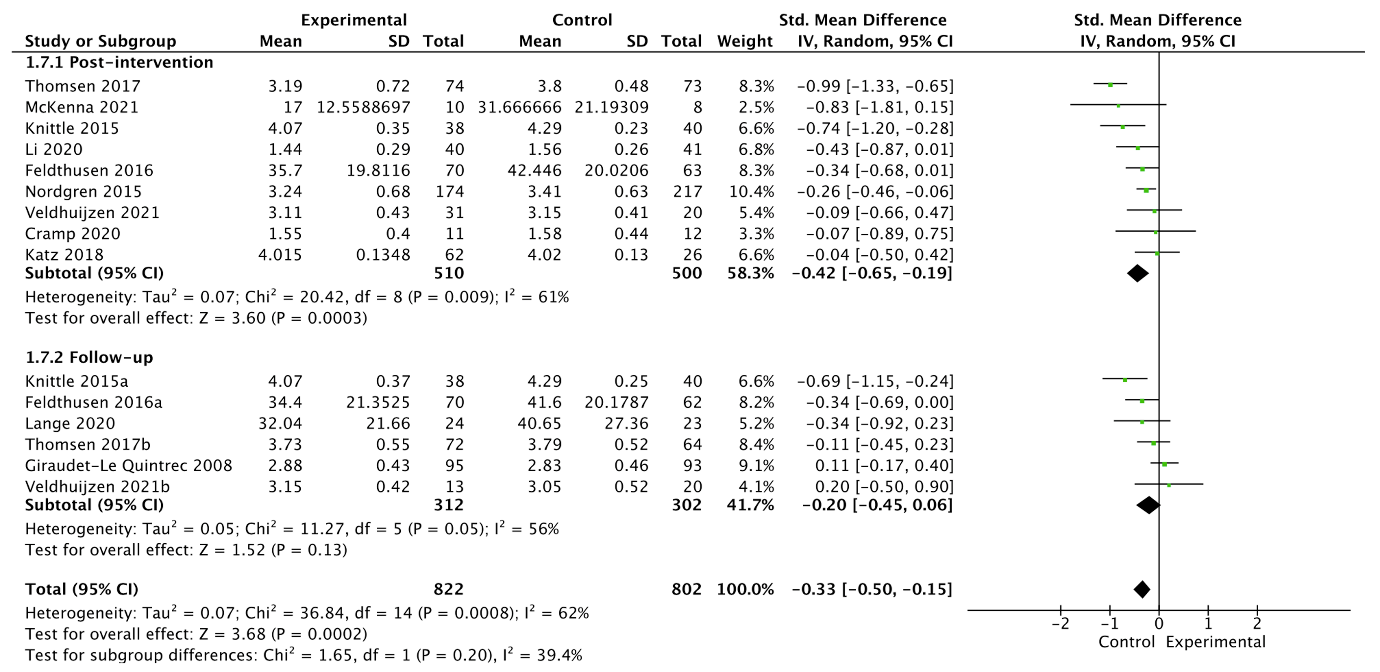


2007

*Supplementary Figure 19: The effects of interventions at post-intervention and follow-up for depression (non-normal). SD= standard deviation, 95% CI= 95% confidence interval.*


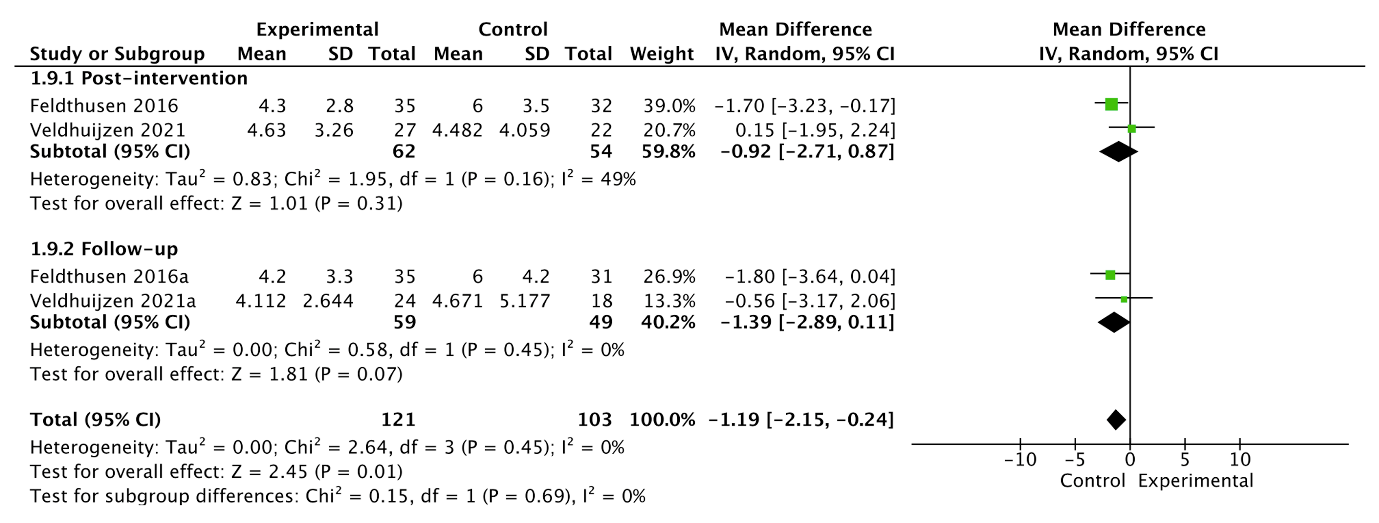


*Supplementary Figure 18: The effects of interventions at post-intervention and follow-up for anxiety. SD= standard deviation, 95% CI= 95% confidence interval.*


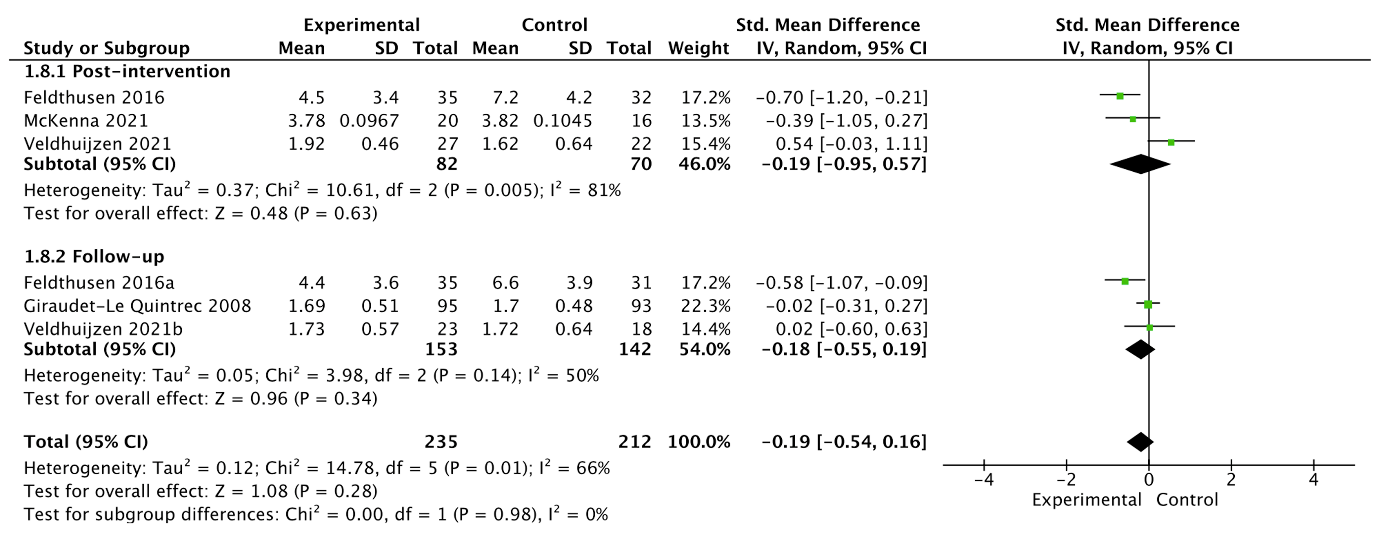


*Supplementary Figure 20: The effects of interventions at post-intervention and follow-up for depression (normally distributed). SD= standard deviation, 95% CI= 95% confidence interval.*


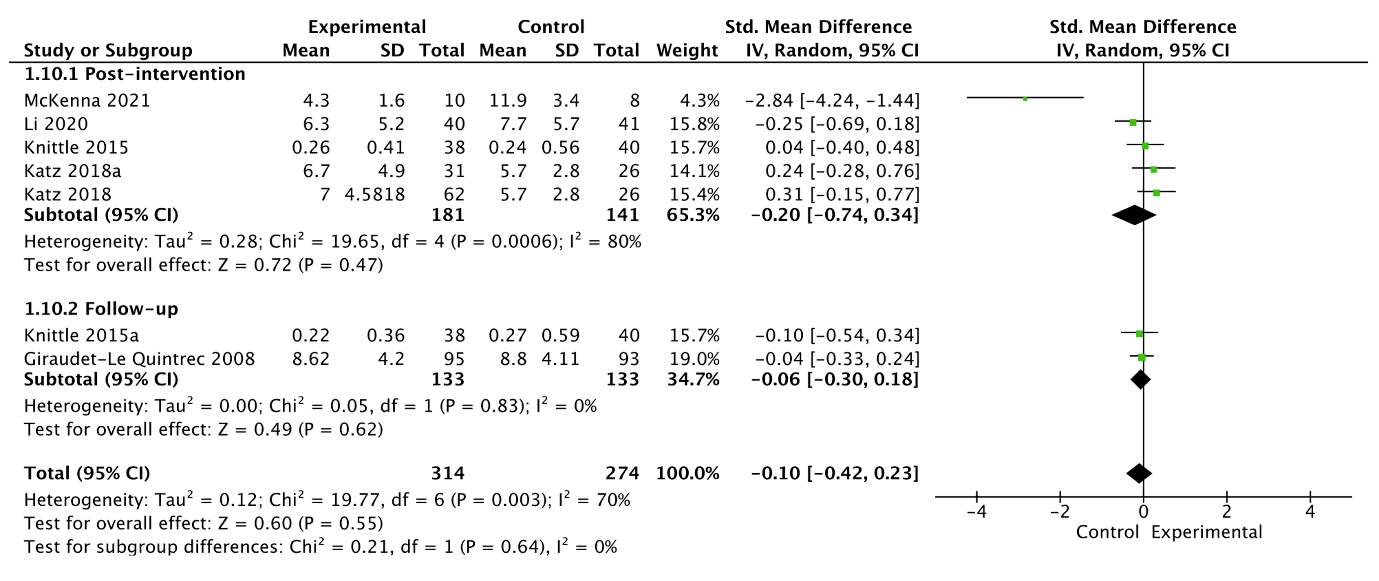


2007

*Supplementary Figure 22: The effects of interventions at post-intervention and follow-up for sedentary time. SD= standard deviation, 95% CI= 95% confidence interval.*


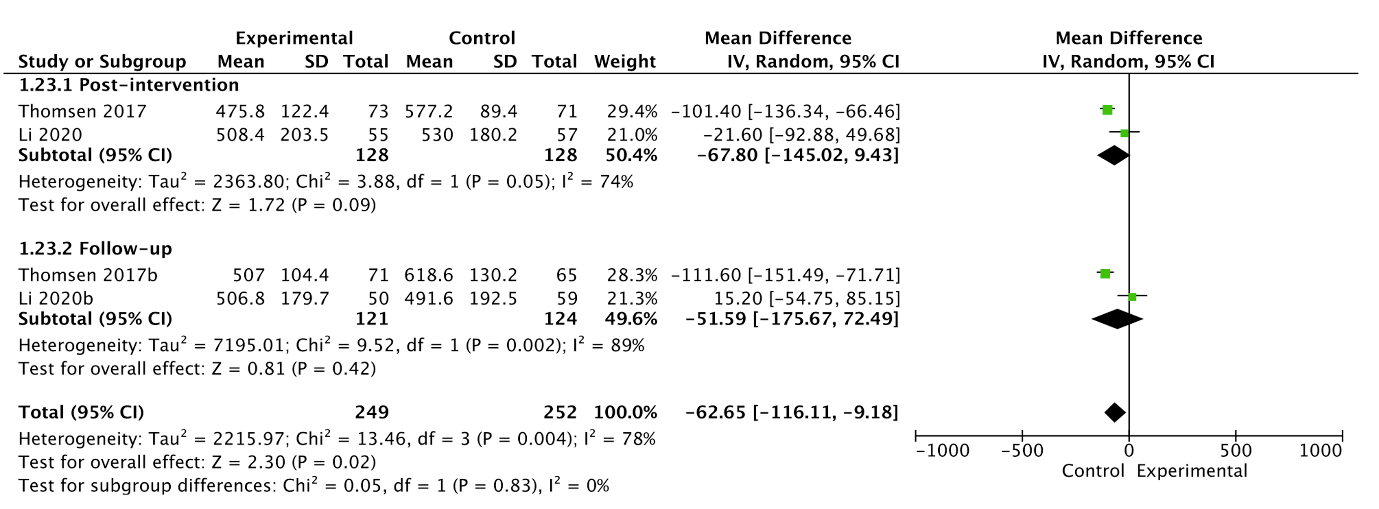


*Supplementary Figure 21: The effects of interventions at post-intervention and follow-up for quality of life. SD= standard deviation, 95% CI= 95% confidence interval.*


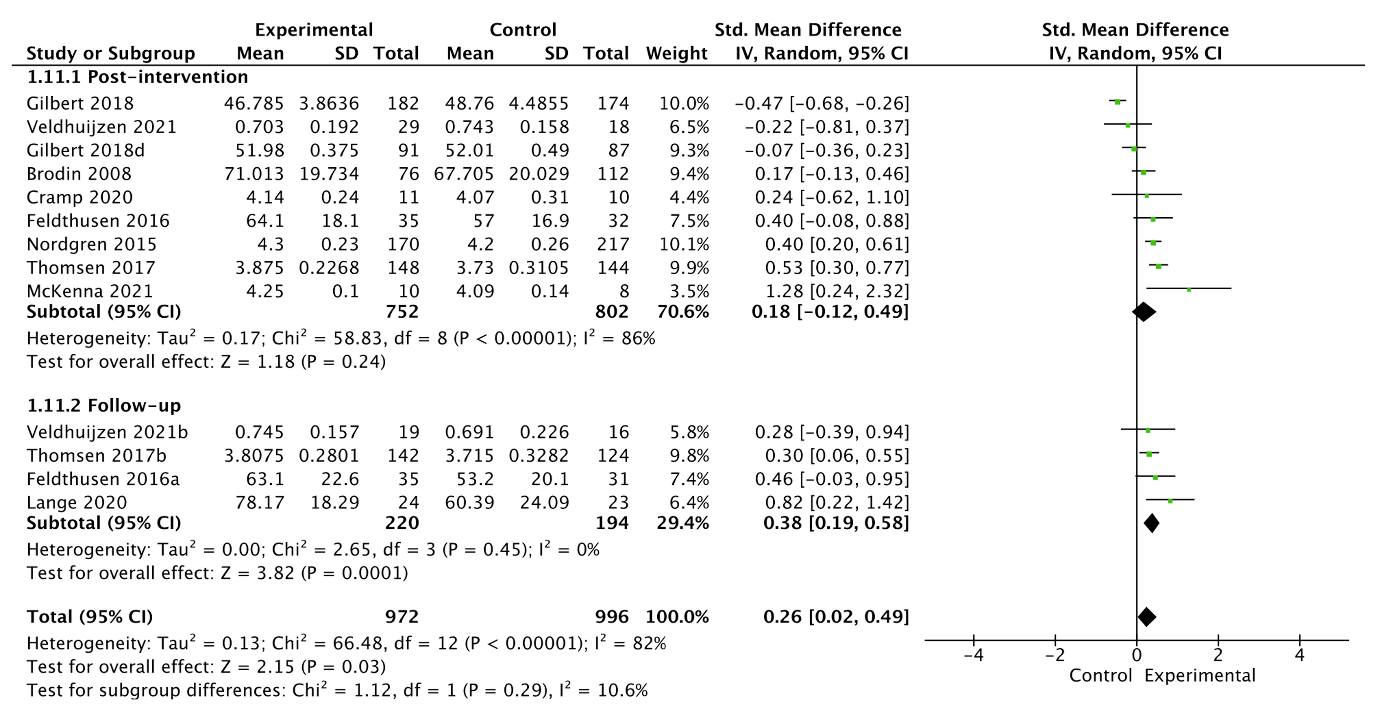


*Supplementary Figure 23: The effects of interventions at post-intervention and follow-up for daily steps. SD= standard deviation, 95% CI= 95% confidence interval.*


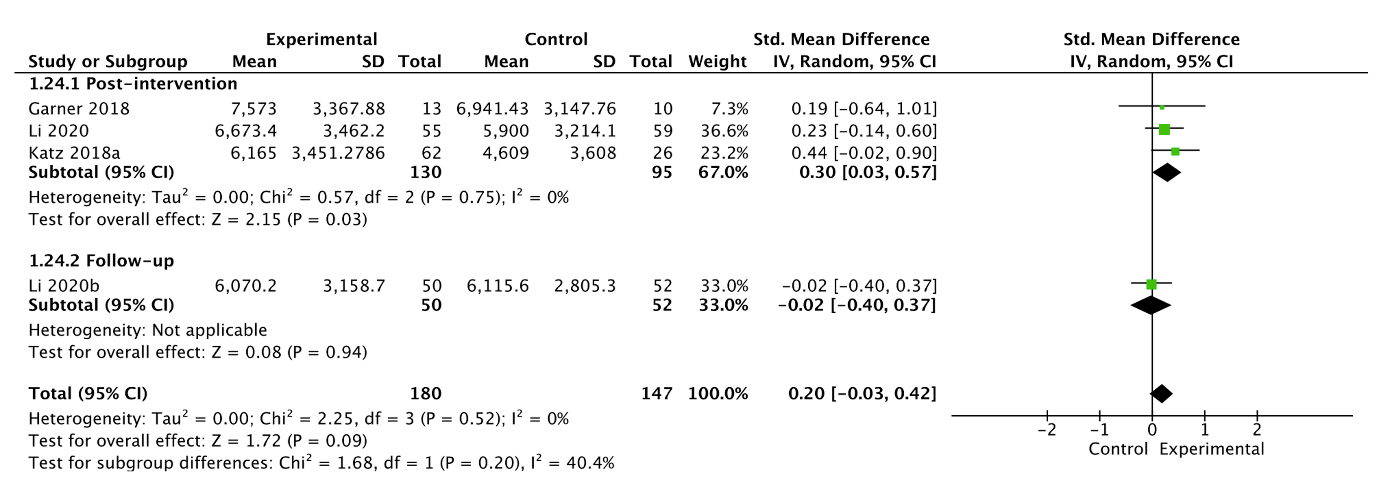


*Supplementary Figure 26: The effects of interventions at post-intervention and follow-up for leisure/light intensity physical activity. SD= standard deviation, 95% CI= 95% confidence interval.*


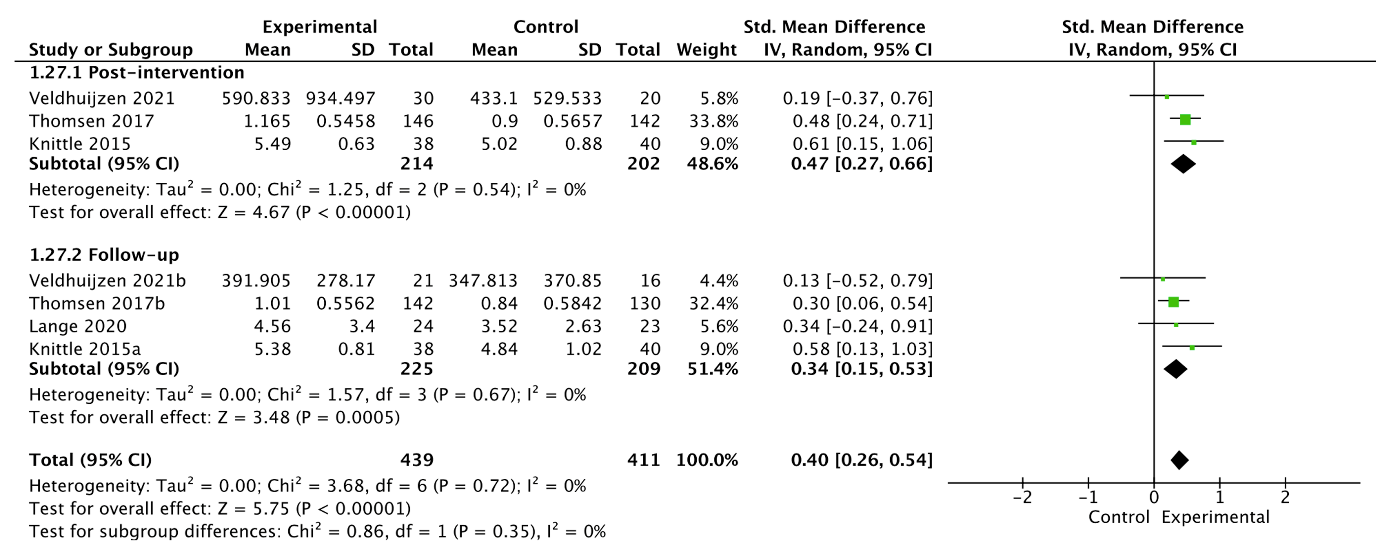


*Supplementary Figure 24: The effects of interventions at post-intervention and follow-up for moderate-to-vigorous physical activity. SD= standard deviation, 95% CI= 95% confidence interval.*


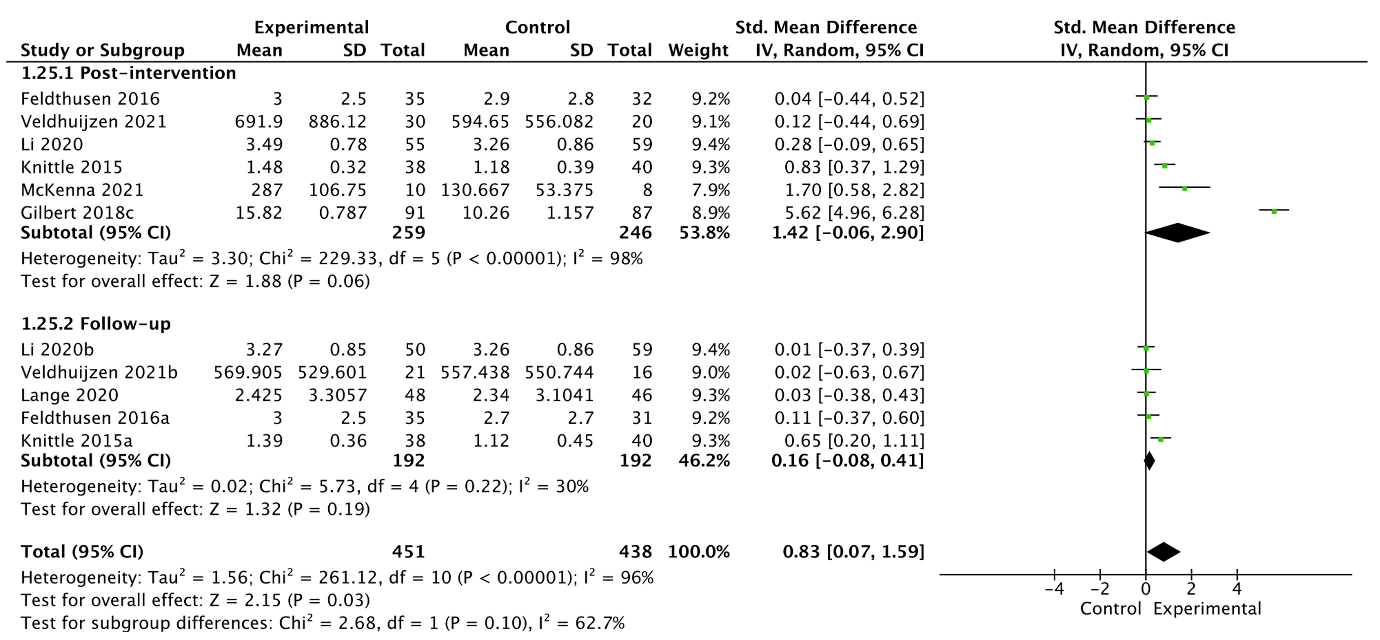


*Supplementary Figure 25: The effects of interventions at post-intervention and follow-up for total physical activity. SD= standard deviation, 95% CI= 95% confidence interval.*


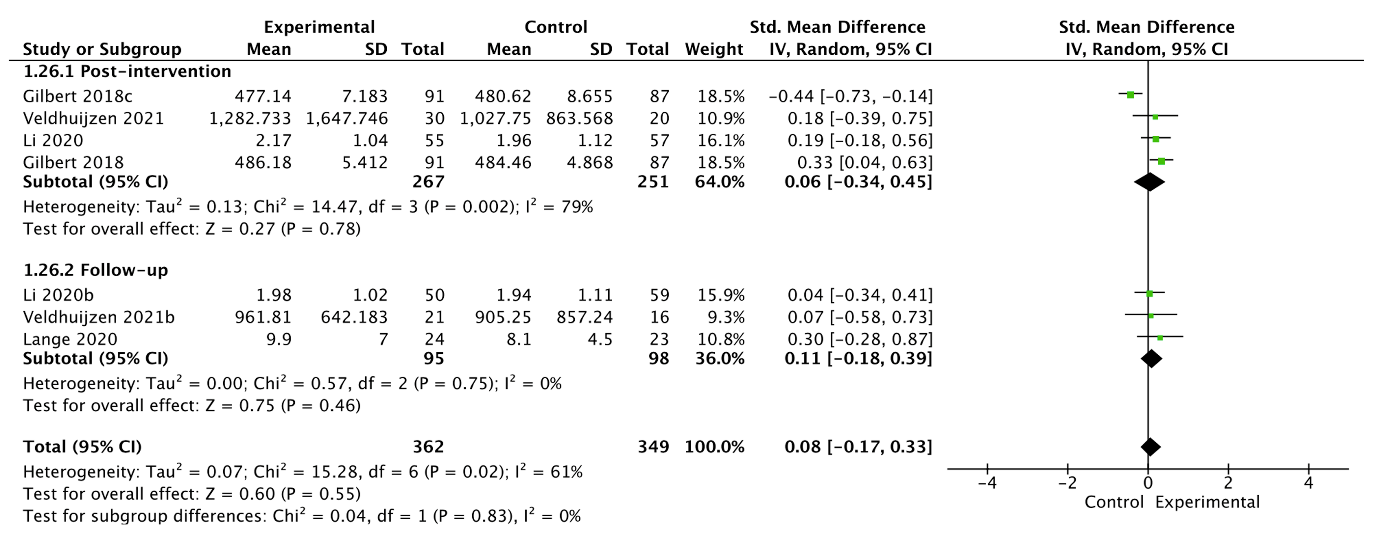


***Supplementary Figures 27-35: Funnel plots for meta-analyses with 10+ entries***

*Supplementary Figure 27: Funnel plot to visualise effects of interventions for disease activity: physical activity vs sedentary behaviour intervention subgroup analysis. SE= standard error, SMD= standardised mean difference*


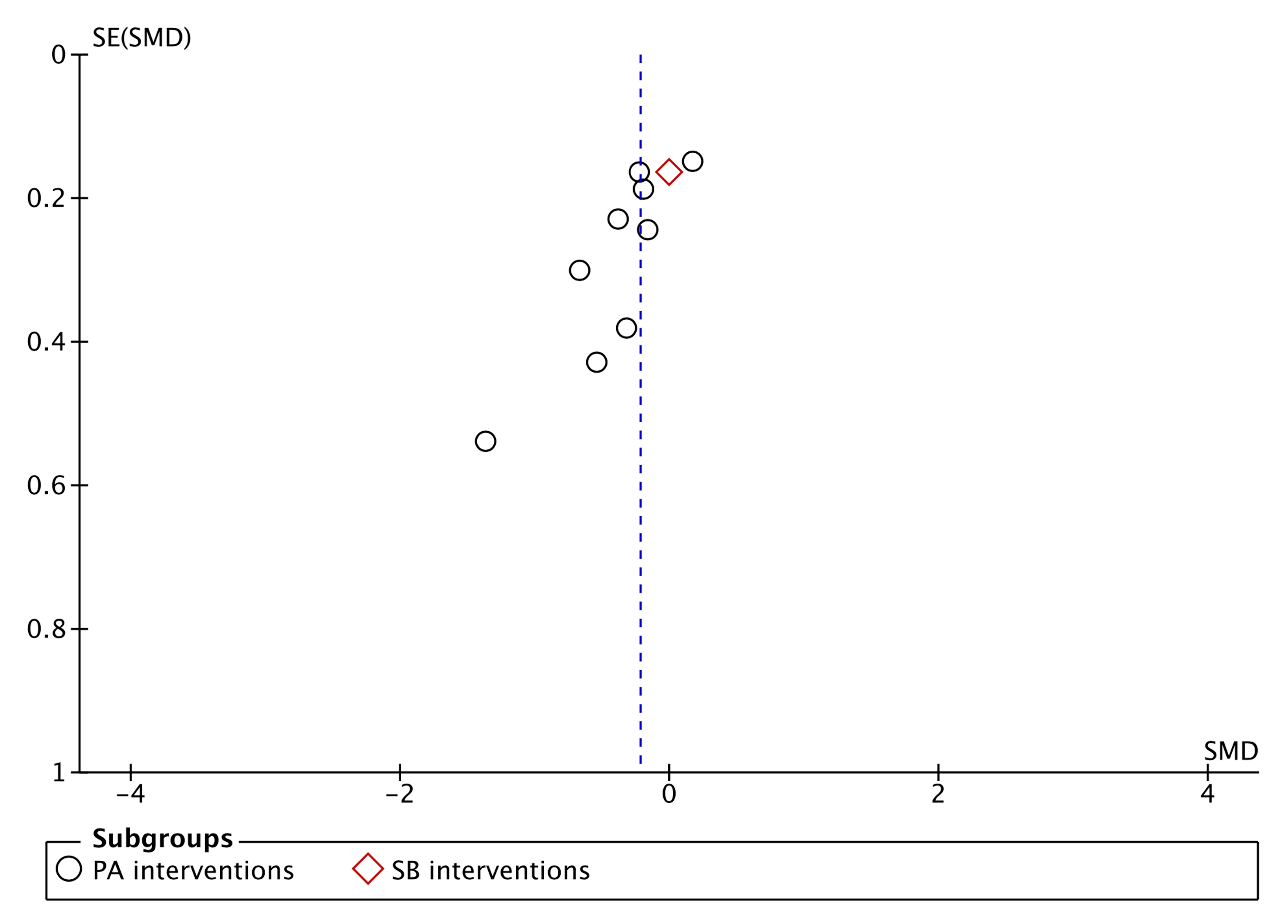


*Supplementary Figure 28: Funnel plot to visualise effects of interventions for disease activity: post-intervention vs follow-up subgroup analysis. SE= standard error, SMD= standardised mean difference*


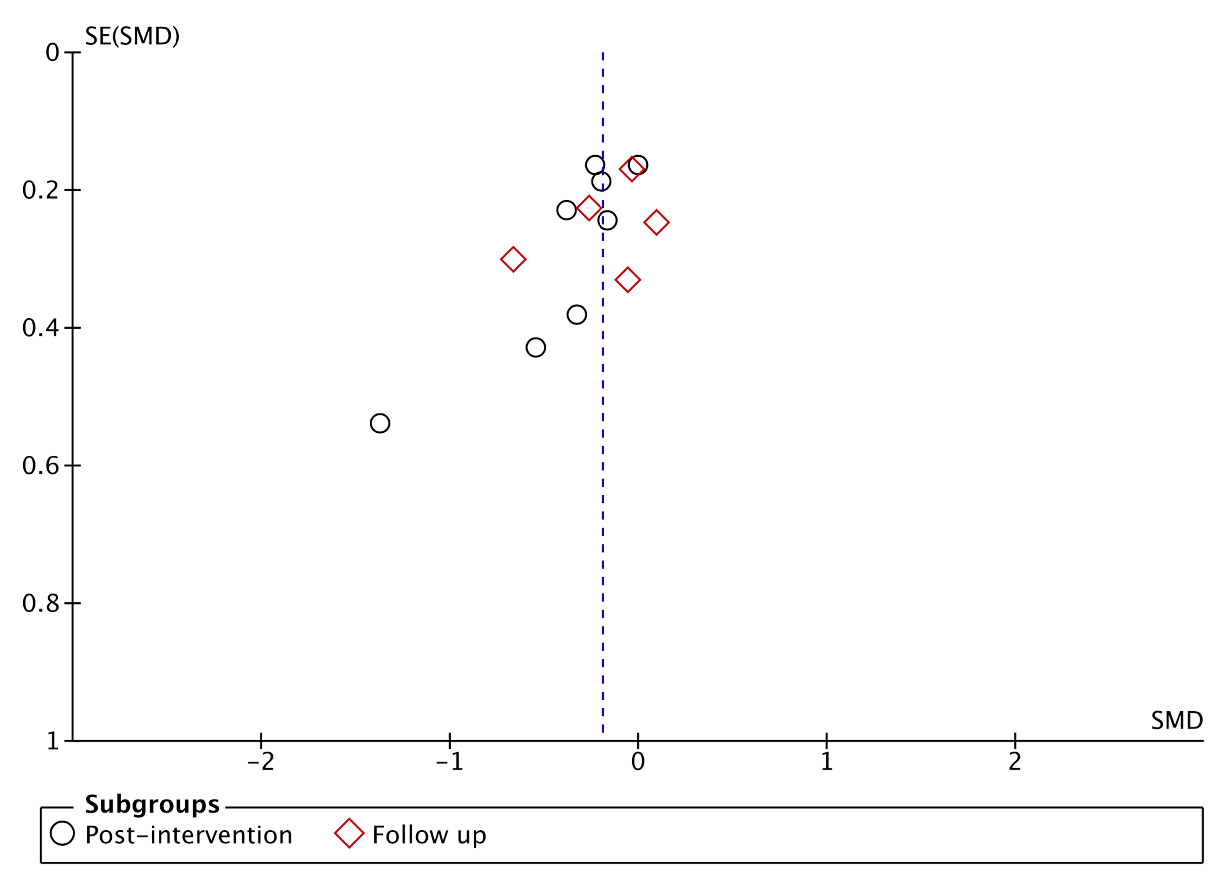


*Supplementary Figure 29: Funnel plot to visualise effects of interventions for pain: physical activity vs sedentary behaviour intervention subgroup analysis. SE= standard error, SMD= standardised mean difference*


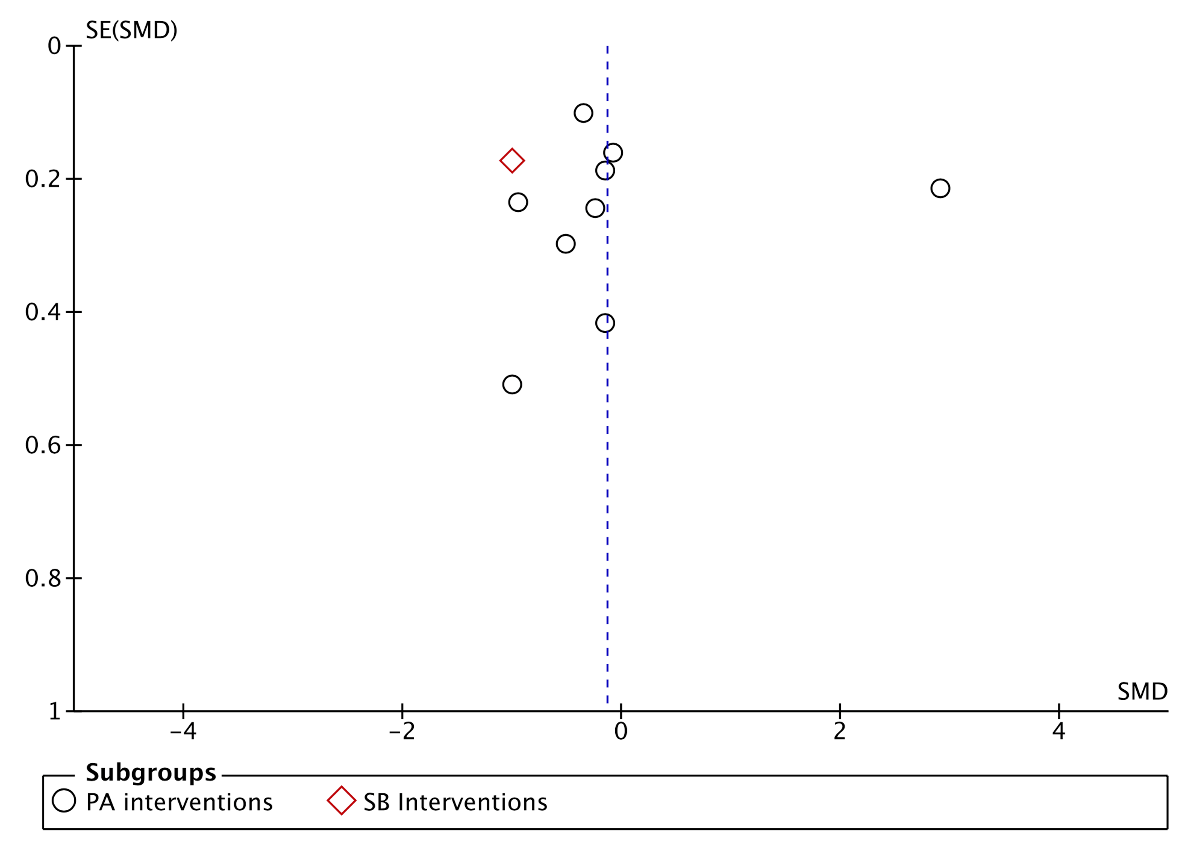


*Supplementary Figure 30: Funnel plot to visualise effects of interventions for fatigue: physical activity vs sedentary behaviour intervention subgroup analysis. SE= standard error, SMD= standardised mean difference*


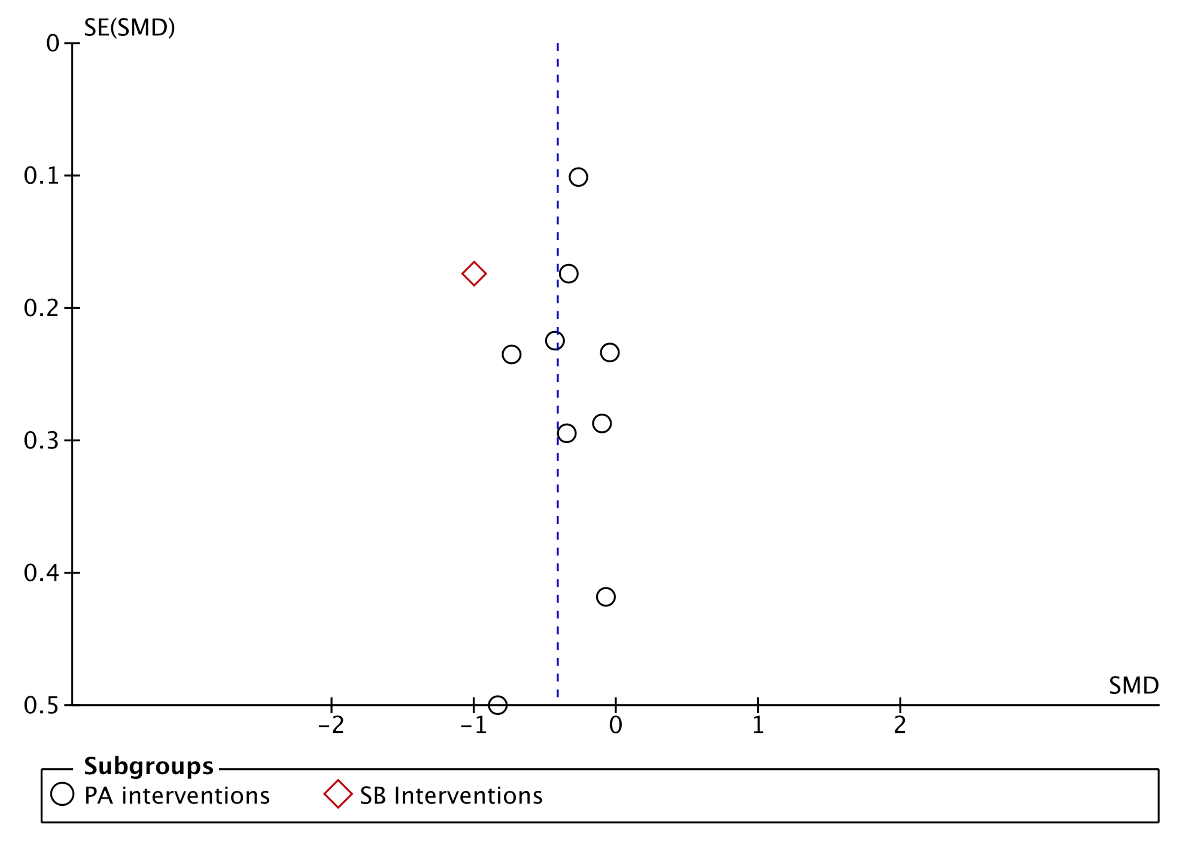


*Supplementary Figure 32: Funnel plot to visualise effects of interventions for pain: post-intervention vs follow-up subgroup analysis. SE= standard error, SMD= standardised mean difference*


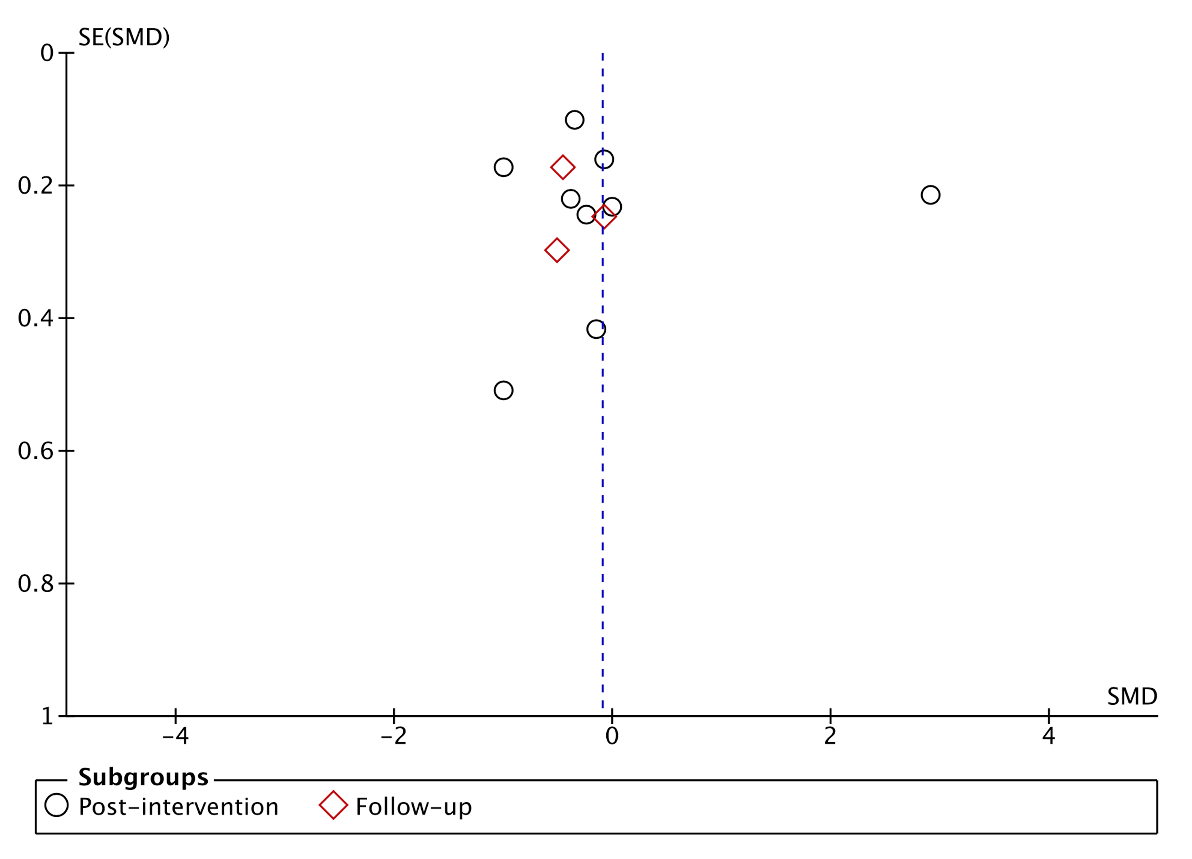


*Supplementary Figure 31: Funnel plot to visualise effects of interventions for functional ability (normally distributed): post-intervention vs follow-up subgroup analysis. SE= standard error, MD= mean difference*


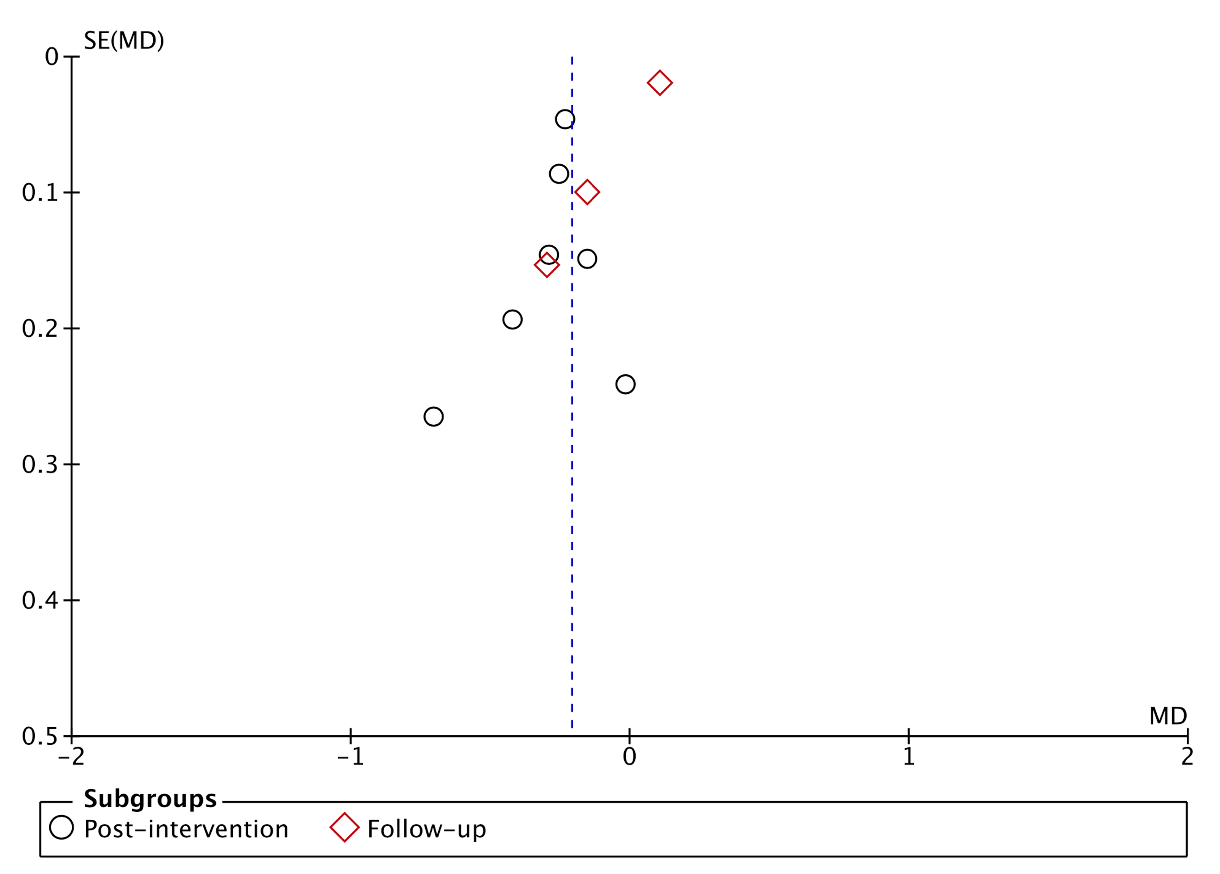


*Supplementary Figure 34: Funnel plot to visualise effects of interventions for quality of life: post-intervention vs follow-up subgroup analysis. SE= standard error, SMD= standardised mean difference*


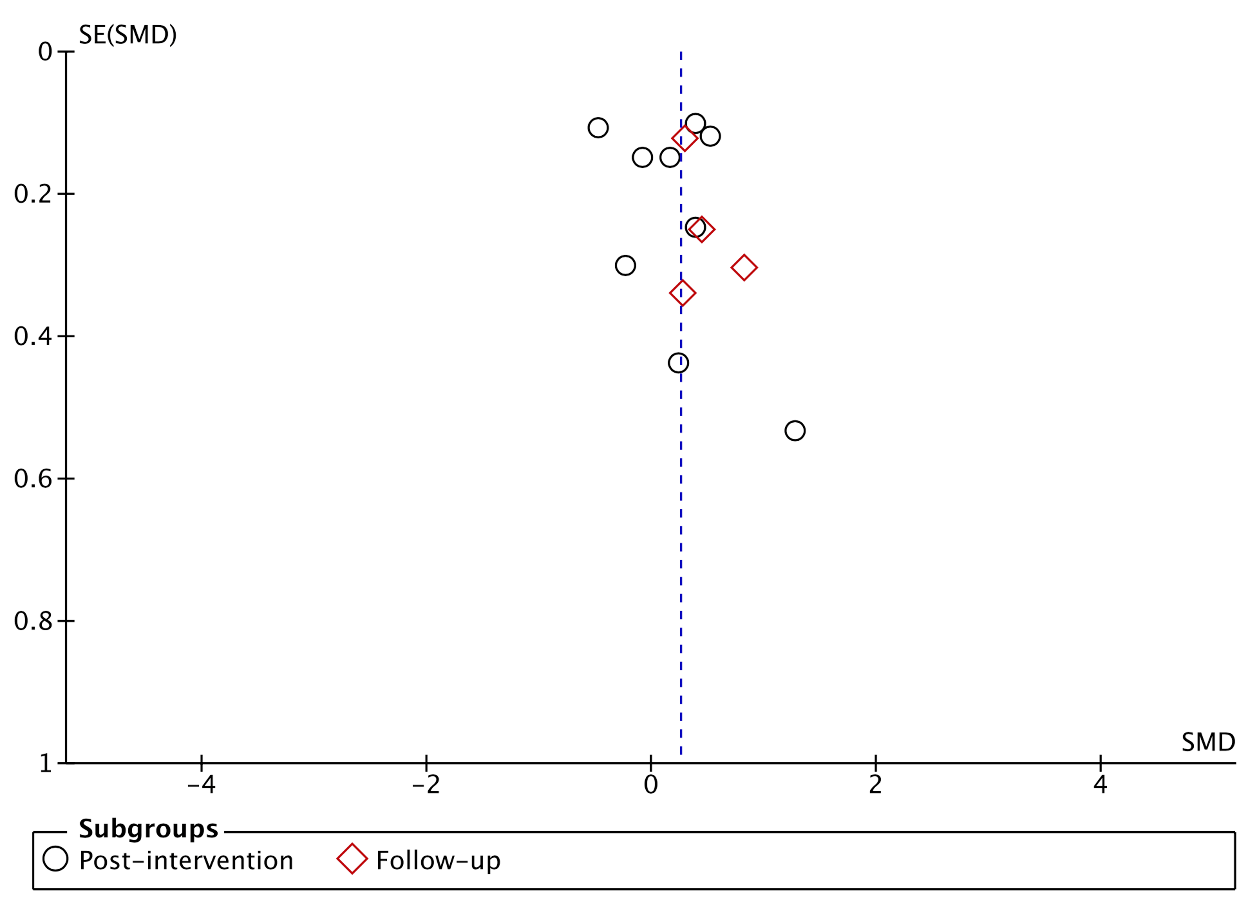


*Supplementary Figure 33: Funnel plot to visualise effects of interventions for fatigue: post-intervention vs follow-up subgroup analysis. SE= standard error, SMD= standardised mean difference*


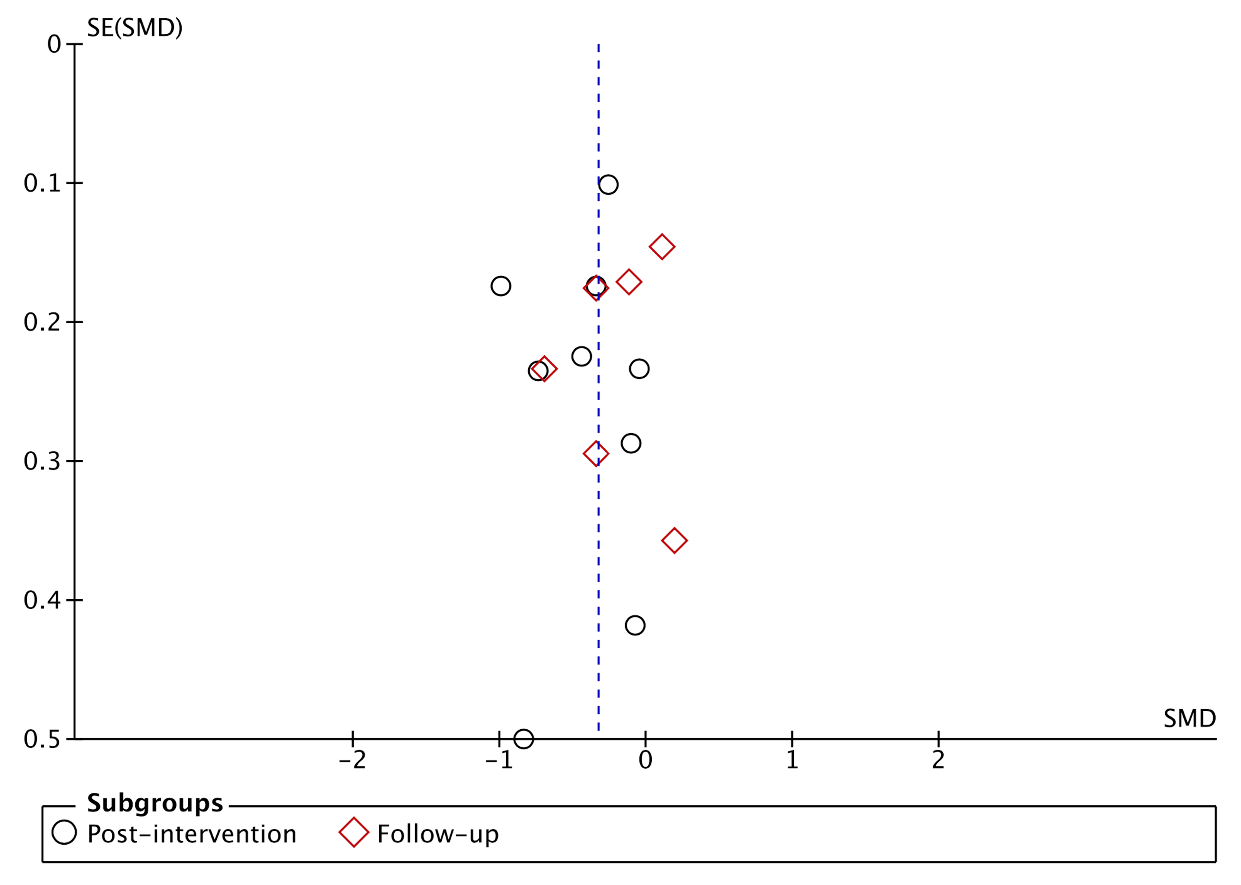


*Supplementary Figure 35: Funnel plot to visualise effects of interventions for moderate-to-vigorous physical activity: post-intervention vs follow-up subgroup analysis. SE= standard error, SMD= standardised mean difference*


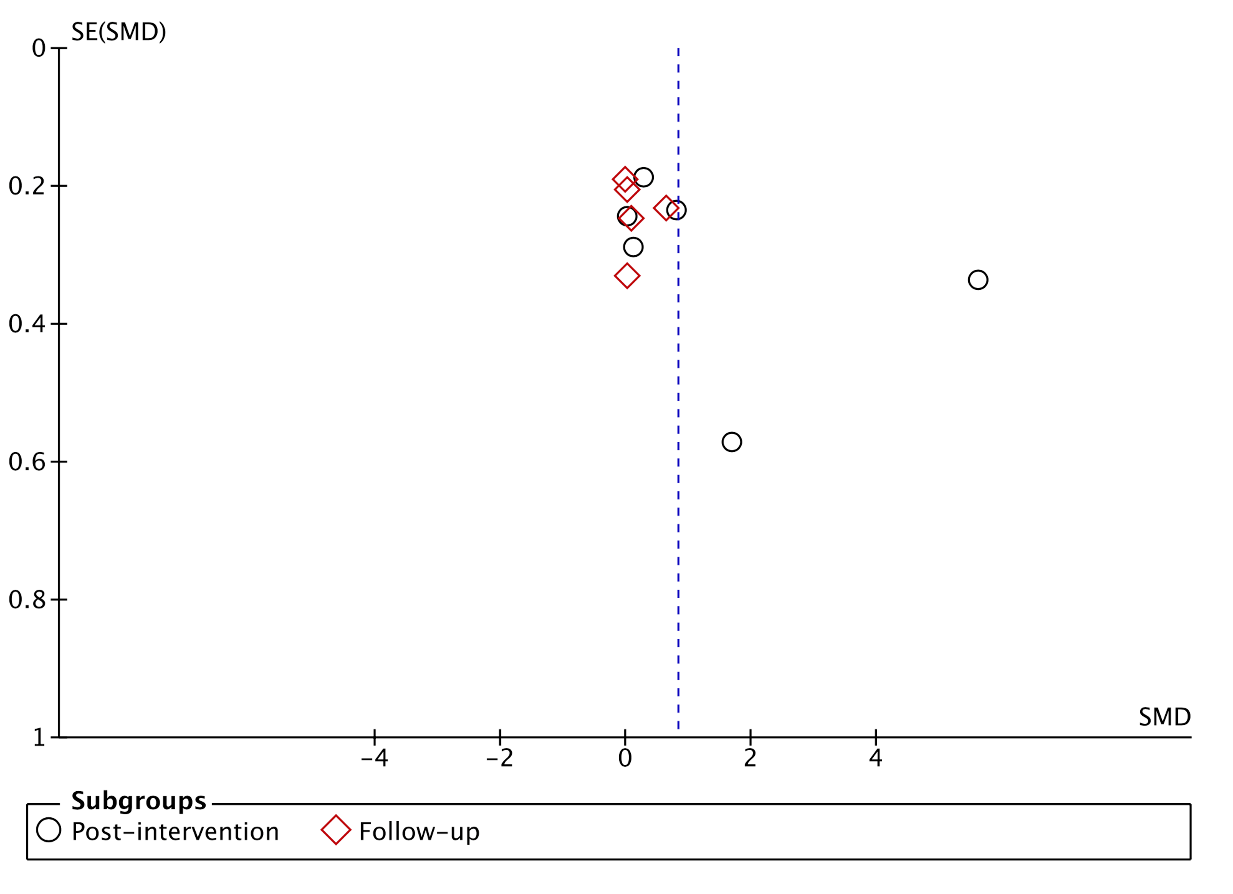

Supplement: Supplementary file 1 — Additional file 1: Supplementary Materials: Supplementary Table 1. PICO question and criteria. Supplementary Table 2. Search Strategies for 8 databases. Supplementary Figures 1-13. Forest plots for secondary outcomes- Physical Activity vs Sedentary Behaviour interventions. Supplementary Figures 14-26. Forest plots for secondary outcomes- Post-intervention vs follow-up. Supplementary Figures 27-35. Funnel plots for meta-analyses with 10+ entries. [file 41927_2023_352_MOESM1_ESM.docx]
